# Importin α1 is required for nuclear import of herpes simplex virus proteins and capsid assembly in fibroblasts and neurons

**DOI:** 10.1371/journal.ppat.1006823

**Published:** 2018-01-05

**Authors:** Katinka Döhner, Ana Ramos-Nascimento, Dagmara Bialy, Fenja Anderson, Ana Hickford-Martinez, Franziska Rother, Thalea Koithan, Kathrin Rudolph, Anna Buch, Ute Prank, Anne Binz, Stefanie Hügel, Robert Jan Lebbink, Rob C. Hoeben, Enno Hartmann, Michael Bader, Rudolf Bauerfeind, Beate Sodeik

**Affiliations:** 1 Institute of Virology, Hannover Medical School, Hannover, Germany; 2 Max-Delbrück Center for Molecular Medicine, Berlin-Buch, Germany; 3 Institute for Biology, University of Lübeck, Lübeck, Germany; 4 Department of Medical Microbiology, University Medical Center Utrecht, Utrecht, The Netherlands; 5 Department of Molecular Cell Biology, Leiden University Medical Center, Leiden, The Netherlands; 6 Research Core Unit Laser Microscopy, Hannover Medical School, Hannover, Germany; Louisiana State University Health Sciences Center, UNITED STATES

## Abstract

Herpesviruses are large DNA viruses which depend on many nuclear functions, and therefore on host transport factors to ensure specific nuclear import of viral and host components. While some import cargoes bind directly to certain transport factors, most recruit importin β1 via importin α. We identified importin α1 in a small targeted siRNA screen to be important for herpes simplex virus (HSV-1) gene expression. Production of infectious virions was delayed in the absence of importin α1, but not in cells lacking importin α3 or importin α4. While nuclear targeting of the incoming capsids, of the HSV-1 transcription activator VP16, and of the viral genomes were not affected, the nuclear import of the HSV-1 proteins ICP4 and ICP0, required for efficient viral transcription, and of ICP8 and pUL42, necessary for DNA replication, were reduced. Furthermore, quantitative electron microscopy showed that fibroblasts lacking importin α1 contained overall fewer nuclear capsids, but an increased proportion of mature nuclear capsids indicating that capsid formation and capsid egress into the cytoplasm were impaired. In neurons, importin α1 was also not required for nuclear targeting of incoming capsids, but for nuclear import of ICP4 and for the formation of nuclear capsid assembly compartments. Our data suggest that importin α1 is specifically required for the nuclear localization of several important HSV1 proteins, capsid assembly, and capsid egress into the cytoplasm, and may become rate limiting *in situ* upon infection at low multiplicity or in terminally differentiated cells such as neurons.

## Author summary

Nuclear pore complexes are highly selective gateways that penetrate the nuclear envelope for bidirectional trafficking between the cytoplasm and the nucleoplasm. Viral and host cargoes have to engage specific transport factors to achieve active nuclear import and export. Like many human and animal DNA viruses, herpesviruses are critically dependent on many functions of the host cell nucleus. Alphaherpesviruses such as herpes simplex virus (HSV) cause many diseases upon productive infection in epithelial cells, fibroblasts and neurons. Here, we asked which nuclear transport factors of the host cells help HSV-1 to translocate viral components into the nucleus for viral gene expression, nuclear capsid assembly, capsid egress into the cytoplasm, and production of infectious virions. Our data show that HSV-1 requires the nuclear import factor importin α1 for efficient replication and virus assembly in fibroblasts and in mature neurons. To our knowledge this is the first time that a specific importin α isoform is shown to be required for herpesvirus infection. Our study fosters our understanding on how the different but highly homologous importin α isoforms could fulfill specific functions *in vivo* which are only understood for a very limited number of host and viral cargos.

## Introduction

Herpesviruses such as herpes simplex virus (HSV), human cytomegalovirus or Epstein-Barr virus cause human diseases ranging from minor ailments to life threatening acute infections, blindness or cancers, particularly in immunocompromised patients. They are complex DNA viruses that depend on many nuclear functions; e.g. triggering the release of the viral genomes from incoming capsids, nuclear import of viral genomes, viral gene expression, genome replication, assembly of progeny capsids, genome packaging into capsids and nuclear capsid egress. Despite these multiple interactions, little is known about the host transport factors that herpesviruses rely on for import through the nuclear pore complexes (NPCs) during infection.

NPCs are the gateways for bidirectional trafficking between cytoplasm and nucleoplasm. The GTPase Ran controls the activity of transport factors to achieve active nuclear import and export of host and viral cargoes. While some import cargoes bind directly to a member of the importin β superfamily, the majority requires one of the importin α isoforms as an adaptor to interact with importin β1. All importin α isoforms share an N-terminal auto-inhibitory importin β1 binding domain followed by a helical core domain of 10 stacked armadillo repeats (ARM), and a small C-terminal acidic cluster; the 7 human importin α isoforms have an amino acid sequence conservation of 42% ([1–4]; reviewed by [5,6]). Classical mono-partite nuclear localization signals (NLSs) utilize a major binding site on ARM 2 to 4, and bipartite NLSs in addition to ARM 2 to 4 a minor binding site on ARM 6 to 8 [7,8]. Furthermore, the C-terminal acidic domain and ARM 9 and 10 contain a third binding site for non-canonical binding motifs [3,9–11]. Different importin α isoforms bind to similar, if not identical NLSs *in vitro*, and their recognition mechanisms are structurally conserved from yeast to human; yet, the affinities to specific importin α isoforms can vary considerably, and they display striking differences in cargo recognition *in vivo* ([2,8,12–14]; reviewed in [5,6]). Importin α links its cargo to importin β1, which in turn binds to NPC proteins to import such ternary complexes into the nucleoplasm, where they disassemble upon interaction with RanGTP (reviewed in [7,15–17]. The nuclear import of several herpesvirus proteins has been shown in transient expression experiments to occur via binding of their NLS to importin α and thus indirectly to importin β1. However, few studies have investigated the specificity of importin α usage *in vitro*, let alone *in vivo* in the context of a viral infection.

Among the herpesviruses, interactions of host nuclear transport factors with viral proteins have been investigated at most for herpes simplex virus type 1 (HSV-1), an alphaherpesvirus that productively replicates in epithelial cells, fibroblasts and neurons. After viral fusion with a host membrane, the incoming capsids utilize dynein for microtubule-mediated transport to the nucleus [18–22]. Capsids covered by inner tegument proteins can bind to the NPCs on nuclei isolated from rat liver or reconstituted from *Xenopus laevis* egg extracts [23,24]. Incoming capsids lacking the large inner tegument protein pUL36 are not targeted to nuclei, and antibodies directed against pUL36 reduce nuclear targeting [25–27]. O’Hare and collaborators have characterized a conserved N-terminal NLS in pUL36 that is essential for targeting incoming capsids to the nucleus and for genome release [28,29]. A likely scenario is that this NLS interacts with host nuclear transport factors to mediate capsid docking to the NPCs. Furthermore, importin β, the RanGTP/GDP cycle and capsid-NPC interactions are required to trigger genome uncoating from capsids; however, a function for importin α could not be uncovered in these *in vitro* assays [23].

HSV-1 promotors in general contain regulatory sequences common with host genes, and are sequentially regulated with immediate-early, early and late gene expression kinetics unless the incoming genomes are repressed and silenced by facultative heterochromatin (reviewed in [30–33]). The tegument viral protein VP16 dissociates from incoming capsids and complexes with the host cell factor HCF-1 and the POU homeodomain protein Oct-1 to keep immediate-early HSV1 promotors de-repressed for transcription (reviewed in [34]). VP16 does not seem to contain an own NLS but piggy-backs onto HCF-1 in the cytosol for co-import into the nucleus; VP16 is not imported into the nucleoplasm, when the NLS in HCF-1 has been mutated [35]. In the nucleoplasm, VP16/HCF binds to Oct-1 that is already associated with HSV-1 promotors [36]. The NLS of Oct-4 interacts with importin α1, Oct-6 with importin α5, while the one of Oct-1 has not been characterized [11,37,38]. In addition to binding sites for VP16, immediate-early HSV-1 promotors also include response elements for the host transcription factors SP1 and GABP [39].

HSV-1 early and late promotors also contain SP1 transcription factor binding sites, and the transcription of viral genes increases after DNA replication due to the increased template number [32,40,41]. The major transactivator ICP4 (infected cell protein 4), the regulators ICP22 and ICP27, and the E3-ubiquitin ligase ICP0 are immediate-early nuclear HSV-1 proteins important for early and late transcription. While their NLSs have been mapped, their nuclear transport factors are not known [42–45]. ICP4 is required for maximal expression from early and late promotors; it recruits the host RNA polymerase II and other host factors, ICP22 and ICP27, and stabilizes the pre-initiation complex [46]. ICP27 is required for efficient viral transcription and translation of some early and early-late genes and perhaps all true late genes. It needs to shuttle between the cytosol and the nucleoplasm to enhance the nuclear export of intron-lacking viral mRNAs and thus their expression (reviewed in [47]). ICP0 also increases the expression of early and late genes; particularly at a low MOI and *in vivo* (reviewed in [48]).

The formation of the nuclear HSV-1 DNA replication compartments results in host chromatin marginalization towards the nuclear rim, and requires seven HSV-1 proteins synthesized with early kinetics. These are the origin-binding protein pUL9, the ssDNA binding protein ICP8 (pUL29), the heterotrimeric helicase-primase complex (pUL5, pUL8, pUL52), and the DNA polymerase with the catalytic subunit pUL30 and its processivity factor pUL42 (reviewed in [49,50]). An NLS of pUL9 has been mapped to its amino acid residues 793 to 804 [51], and the nuclear localization of ICP8 is mediated by its 28 C-terminal amino acid residues [52]. In contrast, the subunits of the primase/helicase complex remain cytosolic when translated in isolation; but their assembly is sufficient to generate an NLS for nuclear import in the case of HSV-1, Epstein-Barr virus, and Kaposi sarcoma herpesvirus [53–55]. The NLSs of the DNA polymerase subunits have been well characterized for HSV-1, the human cytomegalovirus, Epstein-Barr virus and Kaposi sarcoma herpesvirus (reviewed in [56]. Capsid assembly and packaging of the viral genomes also occur in the nucleoplasm, but the major capsid protein VP5, the capsid protein VP23, and the small capsid protein VP26 are not capable of nuclear import on their own [57]. VP5 requires the capsid scaffolding protein VP22a for localization to the cell nucleus [58], and a non-classical NLS of the triplex capsid protein VP19c is responsible for the nuclear import of the other triplex protein VP23 [57,59,60]. Furthermore, the NLSs of pUL15 and pUL33, of the terminase that catalyzes genome packaging into preassembled capsids, have been characterized in detail [61].

Thus although some few direct interactions between host transport factors and viral nuclear proteins have been elucidated, host transport factors required for specific steps in the herpesvirus life cycle have not been identified yet. Considering that herpesviruses rely on so many nuclear functions, we conducted an RNAi screen to identify nuclear transport factors that are relevant for efficient HSV-1 gene expression. Of the 17 host factors that we had targeted, importin β1, importin α1, importin α6, and transportin 1 were required for efficient HSV-1 gene expression while importin 11, importin 8, transportin 3 and importin 9 seemed to repress HSV-1. Our experiments with fibroblasts from knock-out mice or transduced with lentiviral vectors encoding for shRNAs to perturb the expression of specific importin α isoforms showed that efficient nuclear import of the HSV-1 immediate-early proteins ICP4 and ICP0, and the early proteins ICP8 and DNA polymerase required importin α1 and importin α3 but was restricted by importin α4. Furthermore, the assembly of nuclear capsids, capsid egress into the cytoplasm and formation of infectious virions were reduced in the absence of importin α1, while nuclear targeting of incoming capsids, nuclear import of VP16 and of incoming genomes were not impaired. Similarly, when the expression of importin α1 had been silenced in neurons, nuclear targeting of incoming capsids from the somal plasma membrane or the axonal compartment were also not impaired, but the nuclear import of ICP4, HSV-1 gene expression, and the formation of nuclear capsid compartments was prevented. Our data indicate that the nuclear import of several important HSV-1 proteins and thus efficient HSV-1 infection depend specifically on importin α1 in fibroblasts, and even more so in neurons.

## Results

### Specific nuclear transport factors are required for HSV-1 gene expression

**Fig 1:**
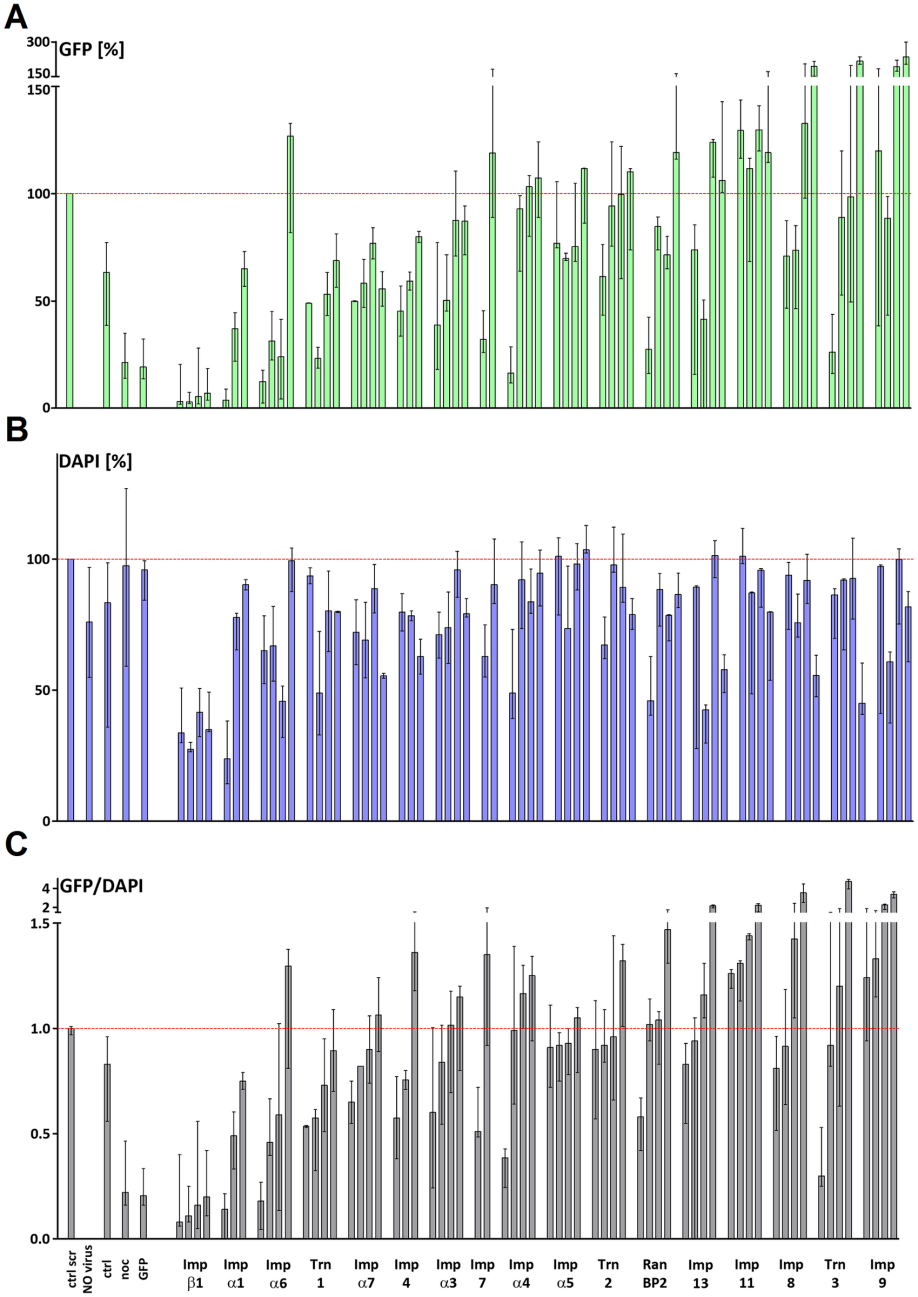
Specific nuclear transport factors are required for HSV-1 early gene expression. HeLaCNX cells were transfected with 50 nM of a scrambled siRNA (scr) or siRNAs directed against different human nuclear transport factors for 72 h and mock infected (no virus) or infected with HSV1(17^+^)Lox-_pMCMV_GFP at 4 x 10^6^ pfu/mL for 12 h. Cells were fixed with 3% para-formaldehyde, permeabilized with 0.1% Triton-X100, and stained with DAPI. The GFP and DAPI levels per well were measured with a plate reader. After subtracting the background GFP signal of the mock infected cells, the GFP/well levels (A, top panel) and the DAPI/well levels (B, middle panel) were normalized to cells transfected with scr siRNAs which had been set to 100%, and the GFP/DAPI ratios were calculated and ranked (C, bottom panel). Medians with the interquartile range of at least two independent experiments each performed in quadruplicates (c.f. S1 Table).

To identify nuclear transport factors required for HSV-1 replication, we transfected HeLa cells with specific siRNAs and infected them at 72 hpt (hour post transfection) with the reporter strain HSV1(17^+^)Lox-_pMCMV_GFP which expresses GFP under the control of a murine cytomegalovirus promoter. At 12 hours post infection (hpi), the HSV1-mediated GFP expression (Fig 1A), and the cell density based on DNA staining were measured in a plate reader (Fig 1B). The GFP signals upon transfection of a scrambled siRNA were normalized to 100% and the background signals of a mock-infected control to 0%. An siRNA directed against the GFP transcripts or treatment with nocodazole served as controls, and reduced HSV1-mediated GFP expression by more than 75%, as expected [62,63]. Nocodazole depolymerizes microtubules that are required for efficient transport of incoming capsids to the nuclear pores, and thus for viral gene expression in epithelial cells [18,20,64,65]. Franceschini *et al*. (2014) have developed an algorithm to subtract some off-target effects of siRNAs with promiscuous seed regions [66]. We applied their criteria to our data which resulted in re-calculating the effect of 4 siRNAs on HSV-1 gene expression (c.f. S1 Table, GFP^corr^). Silencing the expression of some nuclear transport factors reduced cell density, particularly in the case of importin β1 (KPNB1), which is involved in many physiological processes [67,68]. We therefore determined the ratios of the GFP^corr^ signals over the DNA signals, and ranked the nuclear transport factors according to a decreasing inhibition of HSV1-mediated GFP expression per cell upon siRNA treatment (Fig 1C; S1 Table).

Individual siRNAs targeting importin β1 (gene KPNB1), importin α1 (KPNA2), importin α6 (KPNA5), or transportin 1 (TNPO1) decreased HSV-1 mediated GFP/DNA expression on average by more than 30%, whereas most siRNAs directed against importin α7 (KPNA6), importin 4 (IPO4), importin α3 (KPNA4), importin 7 (IPO7), importin α4 (KPNA3), importin α5 (KPNA1), transportin 2 (TPNO2), or Ran binding protein 5 (RANBP5) on average had little effect. In contrast, HSV1-mediated GFP expression was markedly increased by some siRNAs aiming at importin 13 (IPO13), importin 11 (IPO11), importin 8 (IPO8), transportin 3 (TPNO3), or importin 9 (IPO9). These data suggested that HSV1-mediated GFP expression in human HeLa cells particularly depended on importin ß1, importin α1, importin α6, and transportin 1, but might have been restricted by the activities of importin 13, importin 11, importin 9, transportin 3, and importin 9. The nuclear transport factors that were required for efficient HSV-1 mediated GFP expression might contribute to (i) the release of the incoming HSV-1 genomes into the nucleoplasm, (ii) the nuclear import of host transcription factors operating on the MCMV promoter, such as NF-ΚB, AP-1, and SP-1, or (iii) the nuclear import of host or viral factors required for HSV-1 DNA replication, since the amount of the GFP reporter protein depends on the copy number of HSV-1 genomes in the nucleus.

### Importin α expression in fibroblasts

Since we had already shown that importin β1 promotes targeting of incoming HSV-1 capsids to NPCs and viral genome uncoating [23], we focused on the next potential hit, importin α1 (KPNA2). Promiscuous siRNA seed regions might result in off-target effects [66], and importin α isoforms are highly homologous; we therefore decided to use murine embryonic fibroblasts (MEFs) derived from specific importin α knock-out mice for functional experiments. Like others, we use the numbering of the human proteins also for their closest murine homologs: importin α1 (hImp α1, gene *KPNA2*; mImp α2, *kpna2*) and importin α8 (*KPNA7*; *kpna7*) for members of the RCH-family, importin α3 (hImp α3, *KPNA4*; mImp α4, *Kpna4*) and importin α4 (hImp α4, *KPNA3*; mImp α3, *Kpna3*) for the QIP family, and importin α5 (*KPNA1*; *Kpna1*), importin α6 (*KPNA5*; no murine homolog), and importin α7 (*KPNA6*; *Kpna6*) for the SRP family [5,69–72]. Mouse embryonic fibroblasts (MEFs) derived from importin α1 (MEF-Impα1^-/-^), importin α3 (MEF-Impα3^-/-^), or importin α4 (MEF-Impα4^-/-^) [73] knock-out mice lacked the respective importin α proteins while the levels of other importins had not been reduced (S1A Fig). These data validate the specificity of the polyclonal anti-peptide antibodies and the respective MEF lines used in this study.

### Importin α1, importin α3, or importin α4 are not required for nuclear targeting of incoming HSV-1 capsids, for nuclear import of incoming HSV-1 genomes, or for nuclear import of HSV1-VP16

**Fig 2:**
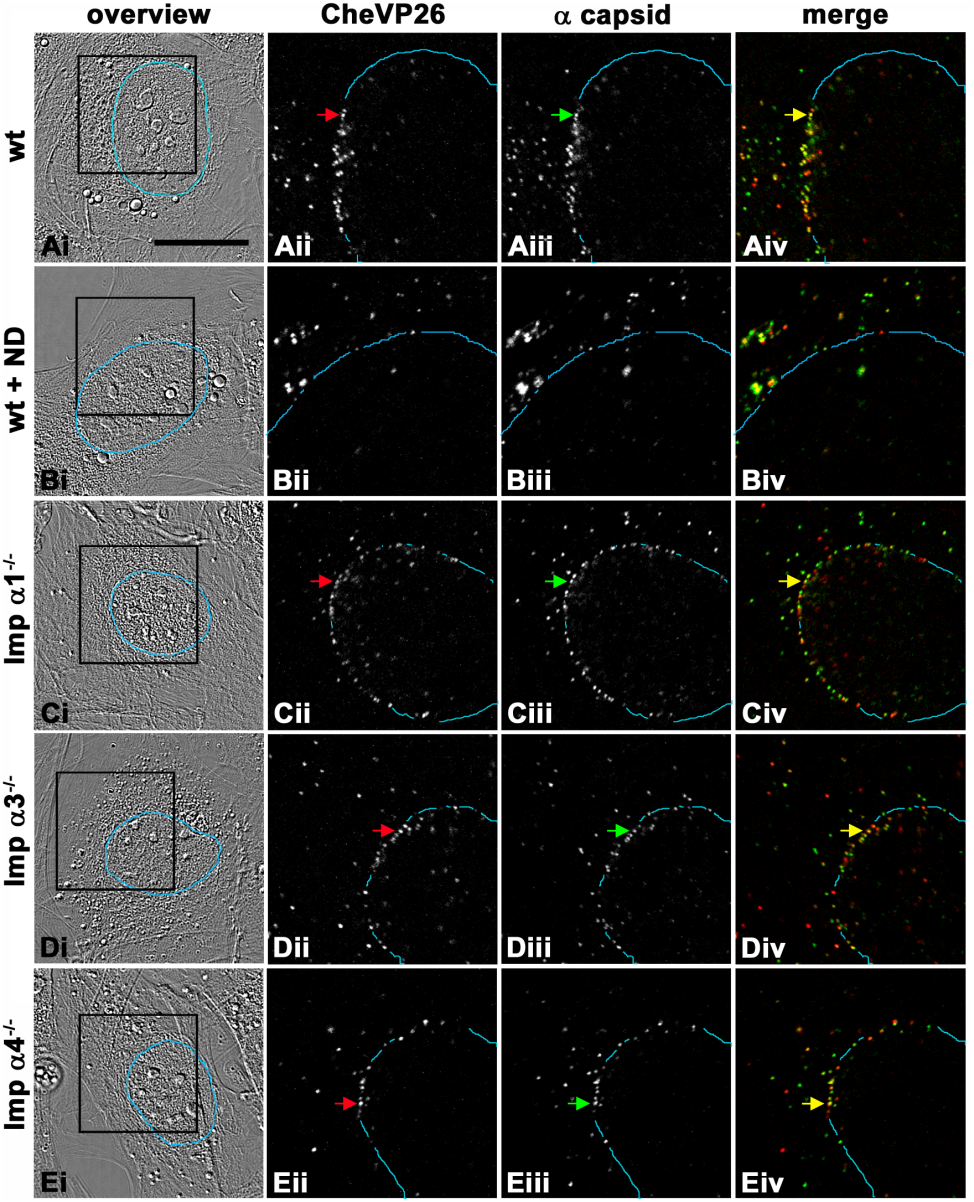
Importin α1, importin α3 or importin α4 are not required for efficient nuclear targeting of incoming HSV-1 capsids. Untreated MEF^wt^ (wt, A), MEF^wt^ treated with nocodazole (wt + ND, B), MEF-Impα1^-/-^ (C), MEF-Impα3^-/-^ (D), or MEF-Impα4^-/-^ (E) were infected with HSV1(17^+^)Lox-CheVP26 (5 x 10^7^ pfu/mL, MOI of 100) in the presence of cycloheximide. The cells were fixed and permeabilized at 4 hpi with PHEMO-fix, labeled with antibodies against capsids (pAb SY4563; iii; green in iv), and analyzed by confocal fluorescence microscopy. CheVP26 was detected by its intrinsic fluorescence (ii, red in iv). Most of the incoming capsids labeled by the α-capsid antibodies and the CheVP26 fluorescence had been targeted to the nuclear rims as determined by DIC (blue lines). The areas boxed in the i panels are shown at higher magnification in the ii, iii, and iv panels. Scale bar: 20 μm.

The first step of the HSV-1 life cycle suggested to recruit an importin α via an NLS is docking of incoming capsids at the NPCs [23–25,28]. We therefore infected MEFs with HSV1(17^+^)Lox-CheVP26 in the presence of cycloheximide to prevent synthesis of progeny HSV-1 proteins, and analyzed the subcellular localization of incoming capsids by confocal fluorescence microscopy. In this HSV-1 reporter strain, the small capsid protein VP26 has been tagged with monomeric Cherry (CheVP26; [74–76]). At 4 hpi, many HSV-1 capsids, detected by CheVP26 (Fig 2Aii; red in Fig 2Aiv) and/or by antibody labeling (Fig 2Aiii, green in Fig 2Aiv), had accumulated at the nuclear rims (Fig 2Ai and blue line Fig 2Aii, 2Aiii and 2Aiv) of MEF^wt^ (Fig 2A). As in epithelial cells [18,20], nocodazole treatment reduced nuclear targeting in MEF^wt^, and instead the capsids were dispersed throughout the entire cytoplasm (Fig 2B). In contrast, incoming capsids accumulated at the nuclear rims of MEF-Impα1^-/-^ (Fig 2C), MEF-Impα3^-/-^ (Fig 2D) or MEF-Impα4^-/-^ (Fig 2E). Thus, HSV-1 internalization into cells and nuclear targeting of incoming capsids were not impaired in MEF-Impα1^-/-^, MEF-Impα3^-/-^, or MEF-Impα4^-/-^.

As efficient HSV-1 gene expression depends on genome uncoating from the capsids and release into the nucleoplasm, we examined the nuclear import of incoming HSV-1 genomes. MEFs were inoculated with HSV1(17^+^) at a high MOI in the presence of cycloheximide, denatured at 3 hpi with an ethanol/acetic acid mixture, and hybridized with a Cy3-labeled DNA probe specific for HSV-1. The cytoplasm and the nuclei of the MEF^wt^ contained many spots of hybridized HSV-1 genomes and mRNAs (S2Aiii Fig). In contrast, there were no signals for HSV-1 in mock-treated cells (S2Biii Fig). The amount of nuclear HSV-1 nucleic acids appeared similar to MEF^wt^ in MEF-Impα1^-/-^ (S2Ciii Fig), MEF-Impα3^-/-^ (S2Diii Fig), and MEF-Impα4^-/-^ (S2Eiii Fig).

Efficient HSV-1 gene expression also depends on nuclear VP16, and we therefore investigated its subcellular localization upon inoculation in the presence of cycloheximide. At 4 hpi, HSV1-VP16 had accumulated to a similar extent in the nuclei of MEF^wt^ (S2Fi Fig), MEF^wt^ treated with nocodazole (S2Gi Fig), MEF-Impα1^-/-^ (S2Hi Fig), MEF-Impα3^-/-^ (S2Ii Fig), and MEF-Impα4^-/-^ (S2Ji Fig). In MEF^wt^ inoculated with the mutant HSV1(17^+^)Lox-ΔgB [77], VP16 had not reached the nucleoplasm as expected, but been retained in virions, located either at the plasma membrane or within endosomes (S2Ki Fig). Glycoprotein B (gB) is essential for HSV-1 cell entry as it catalyzes the fusion of viral with host membranes [78,79]. Consistent with an unimpaired nuclear targeting of incoming capsids, of genomes, and of VP16, we furthermore did not detect any major reorganization of the microtubule network or the distribution of NPC proteins among the different MEF lines (S3 Fig). Taken together, HSV-1 internalization, nuclear targeting of incoming capsids, nuclear import of HSV-1 genomes, and nuclear import of VP16 occurred with similar efficiencies in MEF^wt^, MEF-Impα1^-/-^, MEF-Impα3^-/-^ and MEF-Impα4^-/-^.

### Importin α1 supports and importin α4 restricts efficient HSV-1 protein expression

To determine whether importin α1 is required for viral protein expression, MEF^wt^, MEF-Impα1^-/-^, MEF-Impα3^-/-^, or MEF-Impα4^-/-^ were infected with HSV1(17^+^)Lox and analyzed at 6 hpi by immunoblot. For calibration, we compared the lanes of the knock-out cell lines to lanes in which 25%, 50% or 100% of a comparably infected MEF^wt^ lysate had been loaded (S4 Fig; WT, 25, 50, 100). By 6 hpi, MEF^wt^ and the 3 knock-out lines expressed the immediate-early protein ICP4, the early protein ICP8, and the late tegument proteins VP16 and VP22 (S4A Fig). In contrast, when MEF^wt^ had been inoculated in the presence of nocodazole these proteins were barely detected. A quantitation showed that the expression of ICP4, ICP8 and the late tegument protein VP22 were moderately reduced in the absence of importin α1, but increased in cells lacking importin α4 (S4B Fig). These data indicate that neither importin α1, importin α3, or importin α4 were obligatory but that importin α1 facilitated efficient HSV-1 protein expression while importin α4 restricted it to a certain extent.

### Importin α1 and importin α3 are required for efficient nuclear localization of immediate-early and early HSV-1 proteins

**Fig 3:**
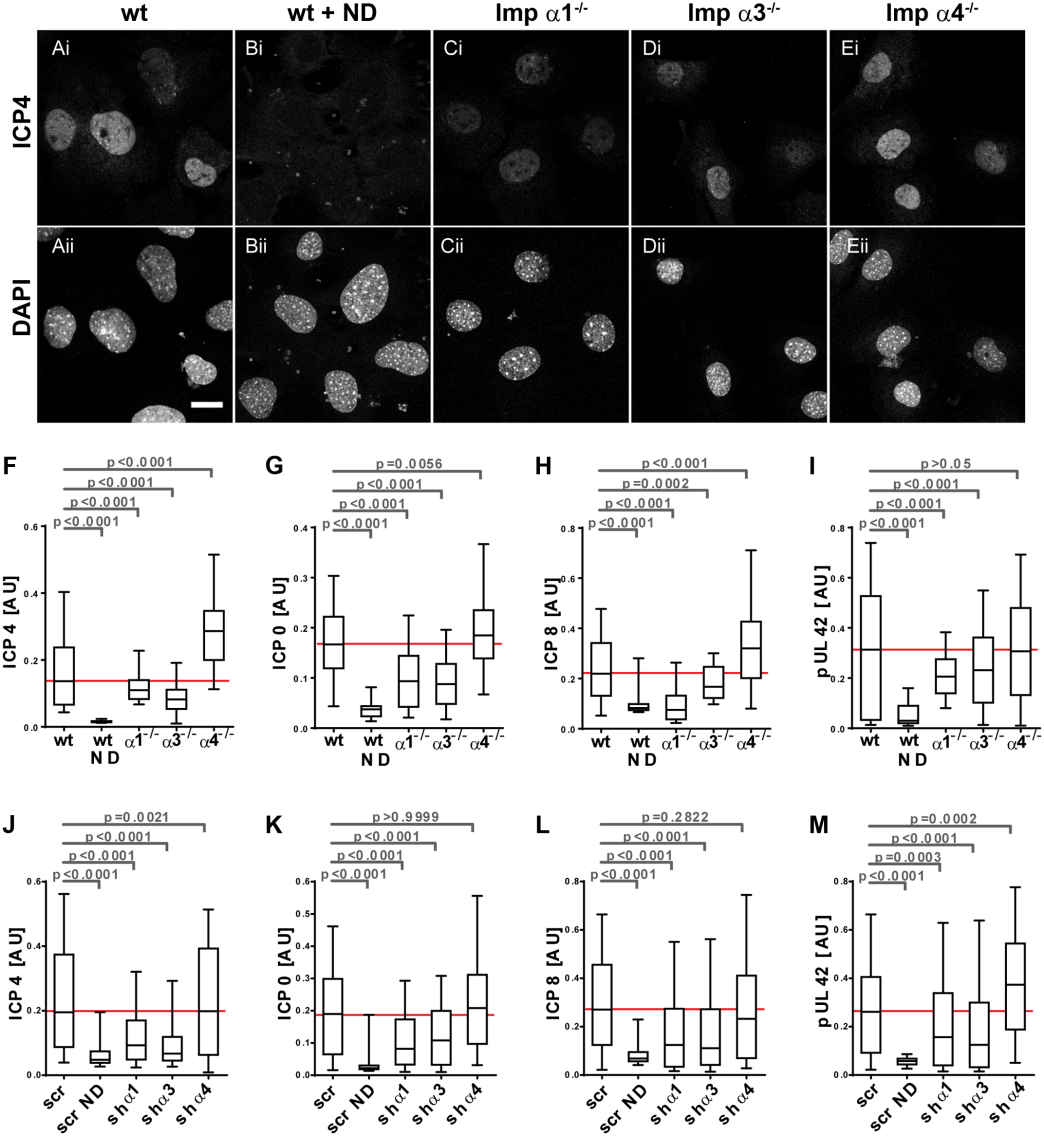
Importin α1 and α3 are required for the nuclear localization of HSV-1 immediate-early and early proteins. (A-E) MEF^wt^ (A), nocodazole treated MEF^wt^ (wt + ND; B), MEF-Impα1^-/-^ (C), MEF-Impα3^-/-^ (D), or MEF-Impα4^-/-^ (E) were infected with HSV1(17^+^)Lox-CheVP26 (0.5 to 1.25 x 10^6^ pfu/mL, MOI of 2 to 5). The cells were fixed at 4, 6 or 8 hpi with 3% PFA, permeabilized with TX-100, labeled for ICP4 (4 hpi; A-E), ICP0 (4 hpi; c.f. S6A–S6E Fig), ICP8 (6 hpi; c.f. S6F–S6J Fig), or pUL42 (8 hpi; c.f. S6K–S6O Fig), and analyzed by confocal fluorescence microscopy. Scale bar 20 μm. (F-I) The mean nuclear fluorescence intensities for ICP4 (F), ICP0 (G), ICP8 (H), or pUL42 (I) were measured in more than 150 randomly selected cells per condition, and are shown as box plots with medians and whiskers representing the 5 to 95% percentile. The p values were determined with a Kruskal-Wallis test followed by Dunn’s multiple comparison testing. (J-M) MEF^wt^ transduced with scr shRNA or shRNAs targeting importin α1, α3 or α4 were infected as described above and the mean nuclear fluorescence intensities for ICP4 (J; c.f. S7A, S7B, S7C, S7D and S7E Fig), ICP0 (K), ICP8 (L; c.f. S7F, S7G, S7H, S7I and S7J Fig) or pUL42 (M) were measured and plotted as described above.

We next determined the impact of different importin α isoforms on the subcellular localization of several HSV-1 proteins required for early gene expression and for DNA replication. MEF^wt^, MEF-Impα1^-/-^, MEF-Impα3^-/-^, or MEF-Impα4^-/-^ were infected with HSV1(17^+^)Lox-CheVP26, labeled for various HSV-1 proteins, stained for DNA, and analyzed by confocal fluorescence microscopy. By 4 hpi, ICP4 was detected in most nuclei of MEF^wt^ although its amount varied considerably among individual cells (Fig 3Ai). After infection of MEF^wt^ in the presence of nocodazole, ICP4 was not detected (Fig 3Bi), whereas in MEF-Impα1^-/-^ (Fig 3Ci) and in MEF-Impα3^-/-^ (Fig 3Di) there was some nuclear ICP4, although considerably less than in MEF^wt^ or MEF-Impα4^-/-^ (Fig 3Ei). A quantification of more than 150 cells for each condition showed that the control nocodazole treatment prevented nuclear localization of ICP4, and that there was significantly less nuclear ICP4 in MEF-Impα1^-/-^ and in MEF-Impα3^-/-^, but more in MEF-Impα4^-/-^ when compared to MEF^wt^ (Fig 3F). Similar results were obtained for ICP0 (S5 Fig). Infection in the presence of nocodazole had also prevented ICP0 expression (S5B Fig), and there was less nuclear ICP0 in MEF-Impα1^-/-^ (S5Ci Fig) and in MEF-Impα3^-/-^ (S5Di Fig), but not in MEF-Impα4^-/-^ (S5Ei Fig) when compared to MEF^wt^ (S5Ai Fig). The quantification confirmed that the nuclear localization of ICP0 depended on both importin α1 and importin α3, but not on importin α4 (Fig 3G).

Seven HSV-1 early proteins including ICP8 and pUL42 catalyze nuclear viral DNA replication. By 6 hpi, ICP8 was detected in most nuclei of MEF^wt^ although its amount varied also among cells. ICP8 was diffusively distributed over the entire nucleoplasm, but clearly enriched in certain nuclear regions (S5Fi Fig) which are the sites of HSV-1 DNA replication (reviewed in [49,50]). Infection of MEF^wt^ in the presence of nocodazole did not reveal any ICP8 (S5Gi Fig), whereas in MEF-Impα1^-/-^ (S5Hi Fig) and MEF-Impα3^-/-^ (S5Ii Fig), there was some nuclear ICP8, although considerably less than in MEF^wt^ (S5Fi Fig) or MEF-Impα4^-/-^ (S5Ji Fig). Similarly, the amount of nuclear pUL42 was lowered in MEF-Impα1^-/-^ (S5Mi Fig) and in MEF-Impα3^-/-^ (S5Ni Fig) when compared to MEF-Impα4^-/-^ (S5Oi Fig) or MEF^wt^ (S5Ki Fig), and there was very little nuclear pUL42 if the MEF^wt^ had been inoculated in the presence of nocodazole (S5Li Fig). The quantification of these images showed that the nuclear localization of ICP8 was reduced in the absence of importin α1 to a similar level as treatment with nocodazole, and also reduced in the absence of importin α3, but increased without importin α4 when compared to MEF^wt^ (Fig 3H). Similarly, the nuclear localization of pUL42 was also dependent on importin α1 and on importin α3 but not on importin α4 (Fig 3I).

While the MEF cell lines derived from knock-out animals unequivocally did not express the targeted importin α isoform, they may have compensated its absence during passage in cell culture by increased or decreased expression of other isoforms or related transport factors. As an additional approach, we therefore validated lentiviral vectors expressing shRNAs to silence the expression of importin α1, importin α3, or importin α4 without impairing the expression of other importin α isoforms (S1B Fig). We then infected MEF^wt^ transduced with specific shRNAs or a scrambled shRNA with HSV-1 using the same conditions as for the MEF knock-out lines. The nuclear localization of ICP4 (S6A–S6E Fig, Fig 3J), ICP0 (Fig 3K), ICP8 (S6F–S6J Fig, Fig 3L), and pUL42 (Fig 3M) was significantly reduced upon silencing the expression of importin α1 or importin α3. In contrast, silencing importin α4 expression did not affect the nuclear targeting of ICP4, ICP0 or ICP8, but increased the nuclear amounts of pUL42.

In summary, targeting importin α4 with shRNA did not affect the nuclear amounts of three HSV-1 proteins but lead to an increase of nuclear pUL42. Similarly, the nuclear amount of ICP0 and pU42 was similar in MEF-Impα4^-/-^ as in MEF^wt^, but increased for ICP4 and ICP8. In contrast, importin α1 and importin α3 were required for efficient nuclear localization of the immediate-early expressed proteins ICP4 and ICP0 and the early expressed proteins ICP8 and pUL42.

### Importin α1 is required for nuclear HSV-1 capsid assembly and productive infection

**Fig 4:**
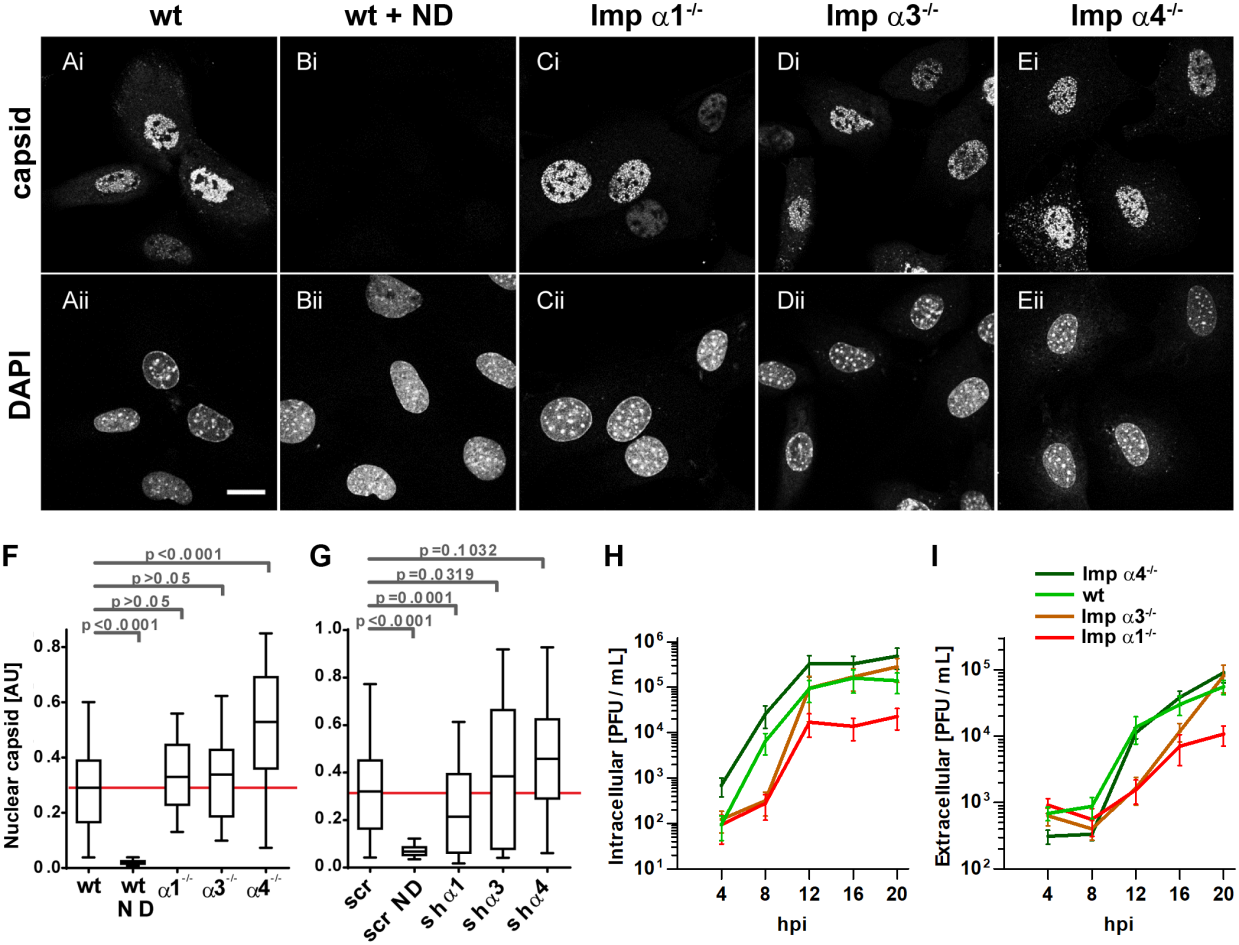
Importin α1 is required for productive HSV-1 infection. **(A-E)** MEF^wt^ (A), nocodazole treated MEF^wt^ (wt + ND, B), MEF-Impα1^-/-^ (C), MEF-Impα3^-/-^ (D), or MEF-Impα4^-/-^ (E) were infected with HSV1(17^+^)Lox-CheVP26 (0.5 to 1.25 x 10^6^ pfu/mL, MOI of 2 to 5), fixed with 3% PFA at 8 hpi, permeabilized with TX-100, labeled with antibodies directed against capsid (pAb SY4563), and analyzed by confocal fluorescence microscopy. Scale bar 20 μm. (F) The mean fluorescence intensities were measured in the nuclear profiles for the capsids in more than 150 randomly selected cells per condition, and are shown as box plots with medians and whiskers representing the 10 to 90% percentile. The p values were determined with a Kruskal-Wallis test followed by Dunn’s multiple comparison testing. (G) MEF^wt^ transduced with scr shRNA or shRNAs targeting importin α1, α3 or α4 were infected, labeled with anti-capsid antibodies, and analyzed by confocal microscopy as described for (F). (H-I) MEF^wt^, MEF-Impα1^-/-^, MEF-Impα3^-/-^, or MEF-Impα4^-/-^ were infected with HSV1(17^+^)Lox (2.5 × 10^6^ pfu/mL, MOI of 10), and cell-associated (H) and extracellular (I) virions were harvested at the indicated time points, and titrated on Vero cells.

As infection progressed to later phases of the viral life cycle, MEF^wt^, MEF-Impα1^-/-^, MEF-Impα3^-/-^, or MEF-Impα4^-/-^ infected with HSV1(17^+^)Lox-CheVP26 were analyzed for nuclear capsid compartments. By 8 hpi, the nuclei of MEF^wt^ (Fig 4Ai), MEF-Impα1^-/-^ (Fig 4Ci), MEF-Impα3^-/-^ (Fig 4Di), and MEF-Impα4^-/-^ (Fig 4Ei) contained prominent amounts of nuclear capsid proteins but no nuclear capsid proteins were detected upon infection in the presence of nocodazole (Fig 4Bi). A quantitation showed that the amount of nuclear capsid protein was similar in MEF^wt^, MEF-Impα1^-/-^, and MEF-Impα3^-/-^, and even increased in MEF-Impα4^-/-^ (Fig 4F). A similar experiment with MEF^wt^ transduced with specific or scrambled shRNAs indicated a moderate reduction in the amount of nuclear capsid protein upon silencing importin α1 expression but no changes in the absence of importin α3 or α4 (Fig 4G).

However nuclear import of capsid proteins does not necessarily indicate proper nuclear capsid assembly. Consistent with an impairment of nuclear events during infection, the production of cell-associated infectious HSV-1 particles was reduced by one log for MEF-Impα1^-/-^, and delayed for MEF-Impα3^-/-^ (Fig 4H). Accordingly, the release of extracellular infectious virions was also delayed and reduced from MEF-Impα1^-/-^, and delayed from MEF-Impα3^-/-^ when compared to MEF^wt^ (Fig 4I).

**Fig 5:**
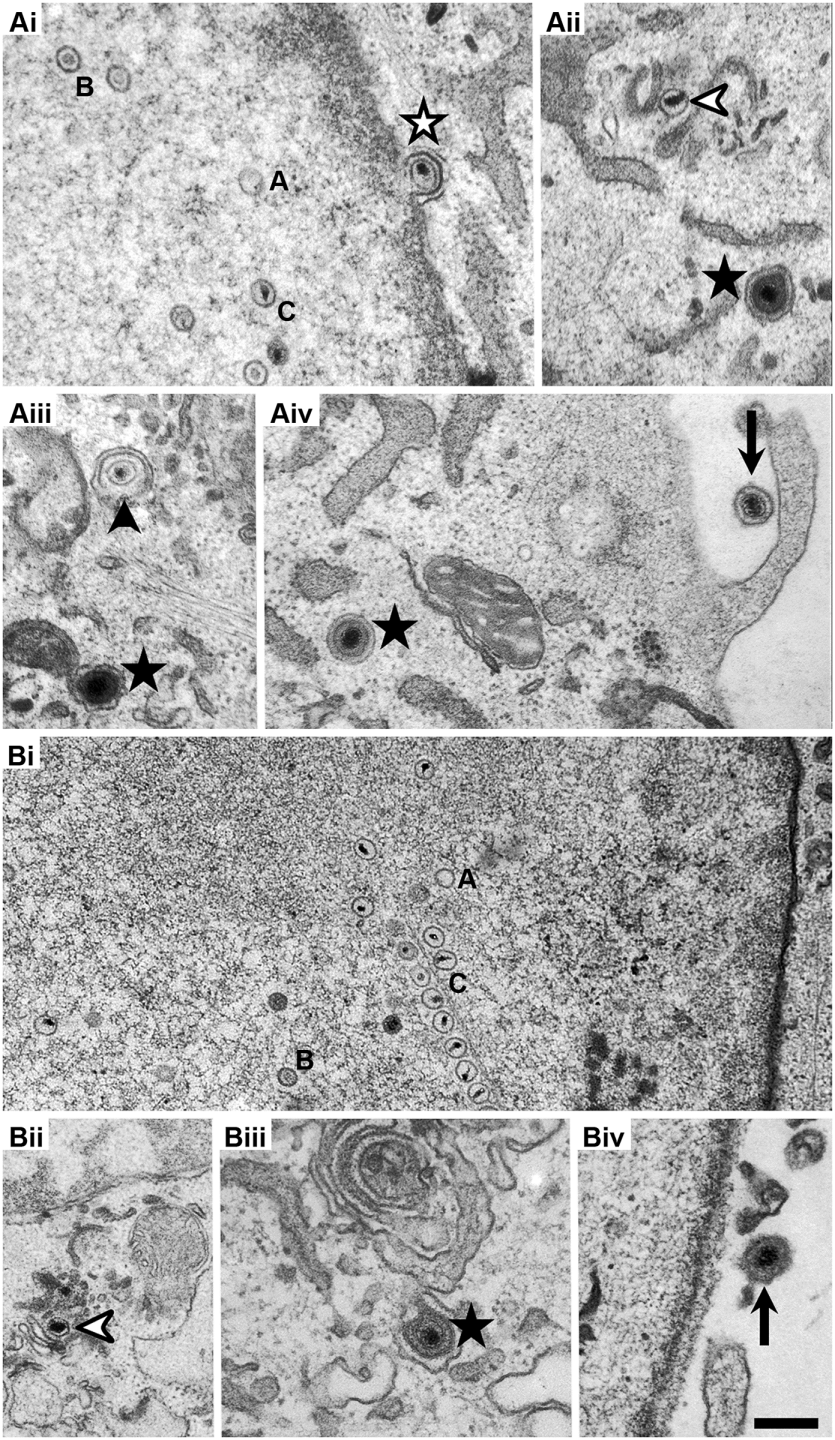
Importin α1 is important for nuclear egress of HSV-1 capsids. MEF^wt^ (A) or MEF-Impα1^-/-^ (B) were infected with HSV1(17^+^)Lox with an MOI of 10 pfu/cell at 2.5 x 10^6^ pfu/mL, fixed at 12 hpi, and analyzed by electron microscopy. In both cell lines, all stages of virus assembly could be identified: nuclear A-capsids (labeled with A in Ai and Bi), B-capsids (labeled with B in Ai and Bi), and C-capsids (labeled with C in Ai and Bi); primary enveloped virions (Ai, white star), cytosolic capsids (Aii, Bii, white arrowheads), wrapping intermediates with capsids being closely associated with cytoplasmic membranes (Aiii, black arrowhead), virions after complete secondary envelopment (Aii, iii, iv, Biii, black stars), and extracellular virions (Aiv, Biv, arrows). Scale bar is 500 nm.

To obtain further insights into capsid and virion assembly, we infected MEF^wt^ (Fig 5A) or MEF-Impα1^-/-^ (Fig 5B) with HSV(17^+^)Lox for 12 h, fixed them, and processed them for analysis by conventional electron microscopy. In both cell types, all known assembly intermediates had been formed: nuclear A, B and C capsids (Fig 5Ai and 5Bi), primary enveloped virions between the inner and the outer nuclear envelope (white star in Fig 5Ai), cytosolic capsids (white arrowhead in Fig 5Aii and 5Bii), capsids in the process of secondary envelopment (black arrowhead in Fig 5Aiii), intracellular vesicles harboring apparently intact virions (black star in Fig 5Aii, 5Aiii, 5Aiv and 5Biii), and extracellular virions attached to the plasma membrane (arrow in Fig 5Aiv and 5Biv). To quantify the amounts of these different assembly intermediates, we systematically evaluated entire cross sections of 10 randomly imaged cells for each cell line (Table 1).

**Table 1:** Importin α1 is required for efficient nuclear egress of progeny capsids.

Cell type		MEF^wt^	MEF-α1^-/-^
**# of cells**		10	10
**Cell area [μm**^**2**^**]**		932	764
**Intracellular capsids / 1,000 μm**^**2**^		634	**449**
**Nuclear area [μm**^**2**^**]**		373	428
**Nuclear capsids / 1,000 μm**^**2**^		1450	**776**
**Nuclear capsids [% of all nuclear]**	**A capsids**	9	4
	**B capsids**	77	55
	**C capsids**	14	**41**
**Cytoplasmic area [μm**^**2**^**]**		559	336
**Cytoplasmic capsids / 1,000 μm**^**2**^		88	**33**
**Cytosolic capsids** [% of cytoplasmic capsids]		27	18
**Wrapping intermediates** [% of cytoplasmic capsids]		63	64
**Enveloped capsids** [% of cytoplasmic capsids]		10	18

MEF^wt^ (left) or MEF-Impα1^-/-^ (right) were infected with HSV1(17^+^)Lox, fixed at 12 hpi, and analyzed by electron microscopy. The number of nuclear A-, B-, and C-capsids; cytosolic capsids, wrapping intermediates with capsids being closely associated with cytoplasmic membranes, and virions after complete secondary envelopment was counted, and the areas of the analyzed nuclear and cytoplasmic regions were measured. While the total number of nuclear and cytoplasmic capsids was reduced in MEF-Impα1^-/-^, the ratio of nuclear C to B capsids was increased.

The amount of intracellular capsids per sampled area was reduced in MEF-Impα1^-/-^ when compared to MEF^wt^. However, although there were fewer nuclear capsids the proportion of nuclear C capsids was increased. In contrast, while there were also fewer cytoplasmic capsids, the relative proportions of the different cytoplasmic capsids, such as cytosolic capsids, capsids in the process of being wrapped by cytoplasmic membranes, and enveloped capsids within transport vesicles was rather similar.

Taken together these observations indicate that importin α1 is required for efficient nuclear capsid assembly and efficient capsid egress. However, those capsids that are translocated into the cytosol seem to associate with cytoplasmic membranes and to become enveloped to a similar extent to form infectious virions that are released from the infected cells also in the absence of importin α1.

### Importin α1 is not required for nuclear targeting of capsids but for HSV-1 gene expression in neurons

**Fig 6:**
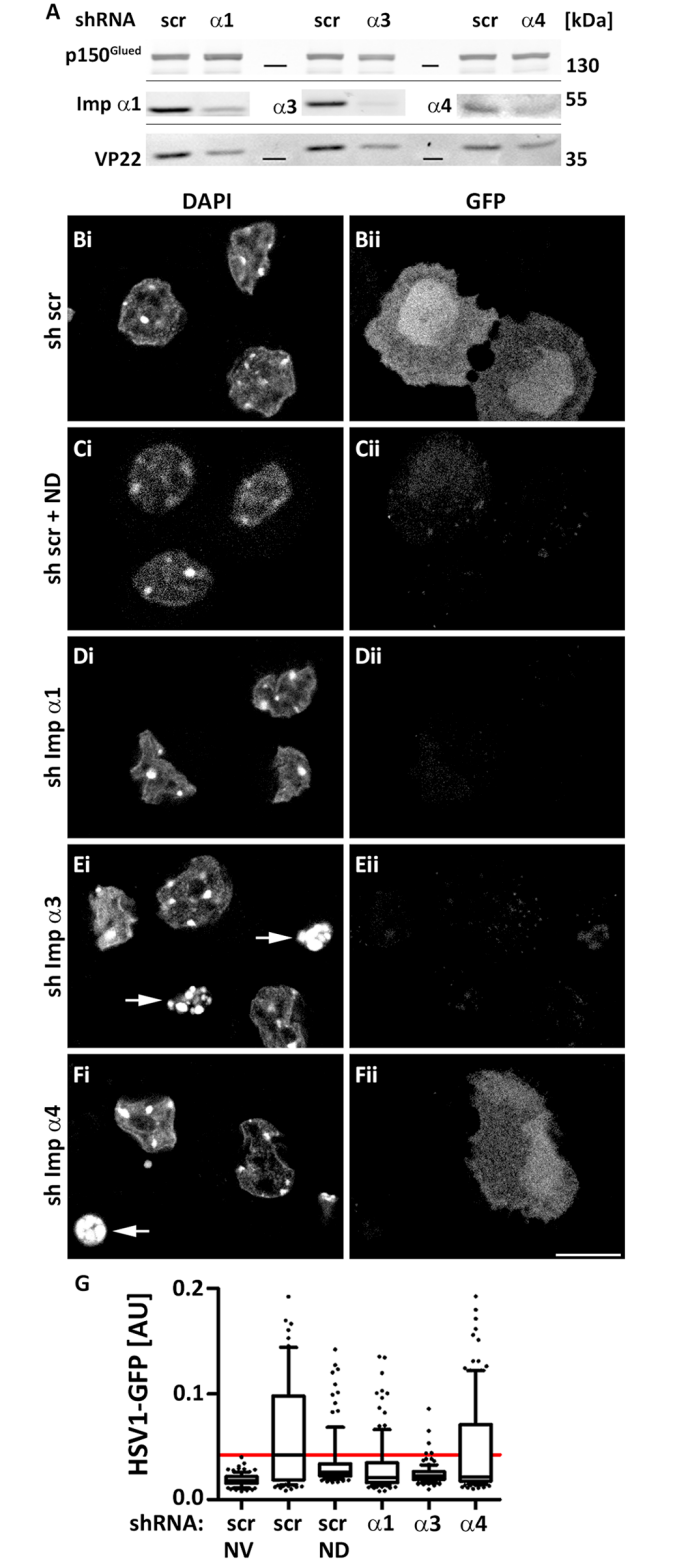
Importins α1 and α3 but to a lesser extent α4 are required for HSV-1 gene expression in neurons. (A) DRG cells cultured in 12-well plates were transduced with scr shRNA or shRNAs against importin α1, α3 or α4 as indicated. At 7 dpt, cells were infected with HSV1(17^+^)Lox-GFP (5 x 10^6^ pfu/mL) for 5 h. Cell lysates were analyzed by immunoblot using antibodies against p150^Glued^, importin α1, α3, α4 or several structural HSV-1 proteins including VP22 (pAb Remus V). (B-F) DRG cells cultured on cover slips were transduced with scrambled shRNA (B, C) shRNA targeting importin α1 (D), α3 (E), or α4 (F). At 7 dpt, the neurons were infected with HSV1(17^+^)Lox-GFP (5 x 10^6^ pfu/mL) in the absence (B, D-F) or presence of 10 μM nocodazole (C; ND). At 4 hpi, the cells were fixed and permeabilized with PHEMO-fix, stained with DAPI (i), and analyzed by confocal fluorescence microscopy. GFP was detected by its intrinsic fluorescence (ii). Scale bar: 10 μm (G) The intra-nuclear GFP signals were quantified with a CellProfiler pipeline using 108 to 129 neurons per condition, and are shown as box plots with medians and whiskers representing the 10 to 90% percentile.

Since importin α isoforms exhibit unique expression profiles in neurons [80], we also investigated the role of importin α in post-mitotic primary neurons derived from the dorsal root ganglia (DRG) of adult mice. We have shown previously that such neurons are susceptible to productive HSV-1 infection [81–83]. We cultured DRG cells for 1 day, transduced them for 7 days with lentiviral vectors expressing an shRNA targeting importin α1, importin α3, importin α4, or expressing a scrambled shRNA, and infected them then with HSV1(17^+^)Lox-GFP. Immunoblotting showed that the expression of the respective importin α isoforms as well of the late tegument protein VP22 was clearly reduced in the DRG cultures when compared to the loading control p150^Glued^, a subunit of the dynein cofactor dynactin (Fig 6A).

**Fig 7:**
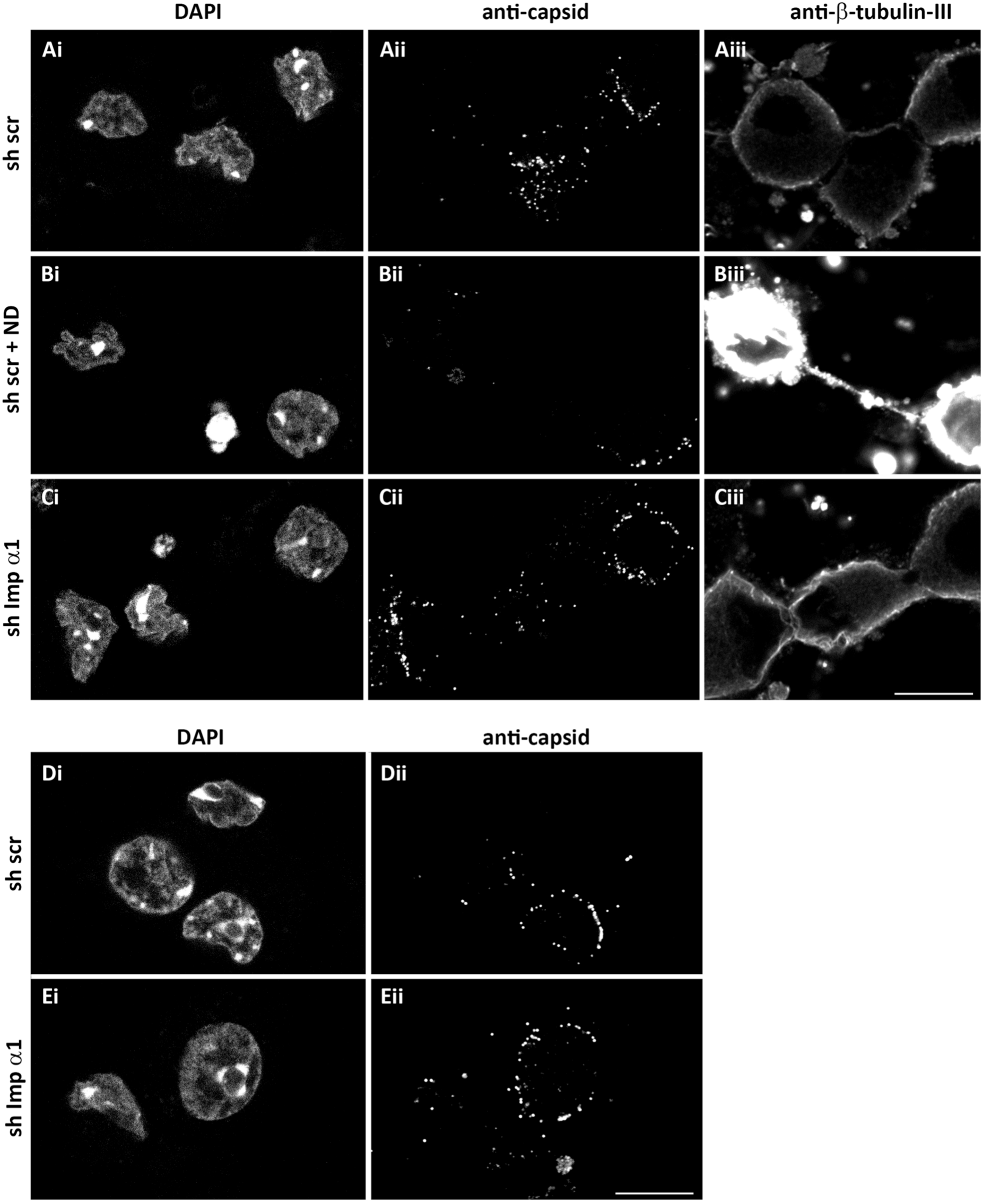
Importin α1 is not required for nuclear targeting of incoming HSV-1 capsids in neurons. (A-C) Primary cells from DRG cultured on cover slips were transduced with scr shRNA (A,B) or shRNA targeting importin α1 (C). At 7 dpt, the cells were infected with HSV1(17^+^)Lox (2.5 x 10^7^ pfu/mL) in the presence of cycloheximide (A, C) or of nocodazole (ND) and cycloheximide (B). At 2.5 hpi, the cells were fixed and permeabilized with PHEMO-fix, stained with DAPI (i), labelled with antibodies against capsid (ii) or β-III-tubulin (iii), and analyzed by confocal fluorescence microscopy. (D-E) DRG cells cultured in microfluidic devices were transduced with scr shRNA (D) or a shRNA against importin α1 (E). At 7 dpt, neurons were selectively inoculated from the axonal side with HSV1(17^+^)Lox-CheVP26 (1.3 x 10^8^ pfu/mL) in the presence of cycloheximide. At 4 hpi, cells were fixed with PFA, stained with DAPI (i), labelled with anti-capsid antibodies (ii), and analyzed by confocal microscopy. Scale bars: 10 μm

We then used confocal fluorescence microscopy to limit our analysis to neurons identified by their typical morphology, their DNA staining pattern (Fig 6Bi–6Fi), and expression of the neuronal β-tubulin-III ([83]; see also Fig 7 below). Neurons expressing scrambled shRNA were well infected as indicated by a prominent HSV-1 mediated expression of GFP (Fig 6Bii). In contrast, there was no GFP detected upon infection in the presence of nocodazole (Fig 6Cii), silencing importin α1 (Fig 6Dii), or silencing importin α3 (Fig 6Eii), while silencing importin α4 did not impair GFP expression (Fig 6Fii). Quantification showed that the levels of nuclear GFP were very heterogeneous among individual neurons and as strongly inhibited in the absence of importin α1 or importin α3 as in the presence of nocodazole (Fig 6G).

We focused the subsequent experiments on the role of neuronal importin α1, since silencing importin α3 often induced changes of the chromatin architecture (arrow in Fig 6Ei). Neurons transduced for shRNA expression were inoculated with HSV-1 in the presence of cycloheximide, fixed at 2.5 hpi, labeled with antibodies against capsids, stained for DNA, and analyzed by confocal fluorescence microscopy. Incoming HSV-1 capsids were as efficiently targeted to the nuclei (Fig 7Ai and 7Ci) of neurons expressing a scrambled shRNA (Fig 7Aii) as after silencing importin α1 (Fig 7Cii). In contrast, nocodazole treatment reduced the number of incoming capsids reaching the neuronal nuclei (Fig 7Bii). Since importin α can contribute to retrograde axonal transport of some cargos [84–87], we also cultured DRG neurons in microfluidic chambers to selectively inoculate the neurons via the axons and not via the plasma membrane of the cell bodies for 4 h. However, nuclear targeting of HSV-1 capsids that in this experimental set-up was strictly dependent on axonal transport was as efficient in neurons expressing a scrambled shRNA (Fig 7Dii) as in neurons silenced for importin α1 expression (Fig 7Eii).

**Fig 8:**
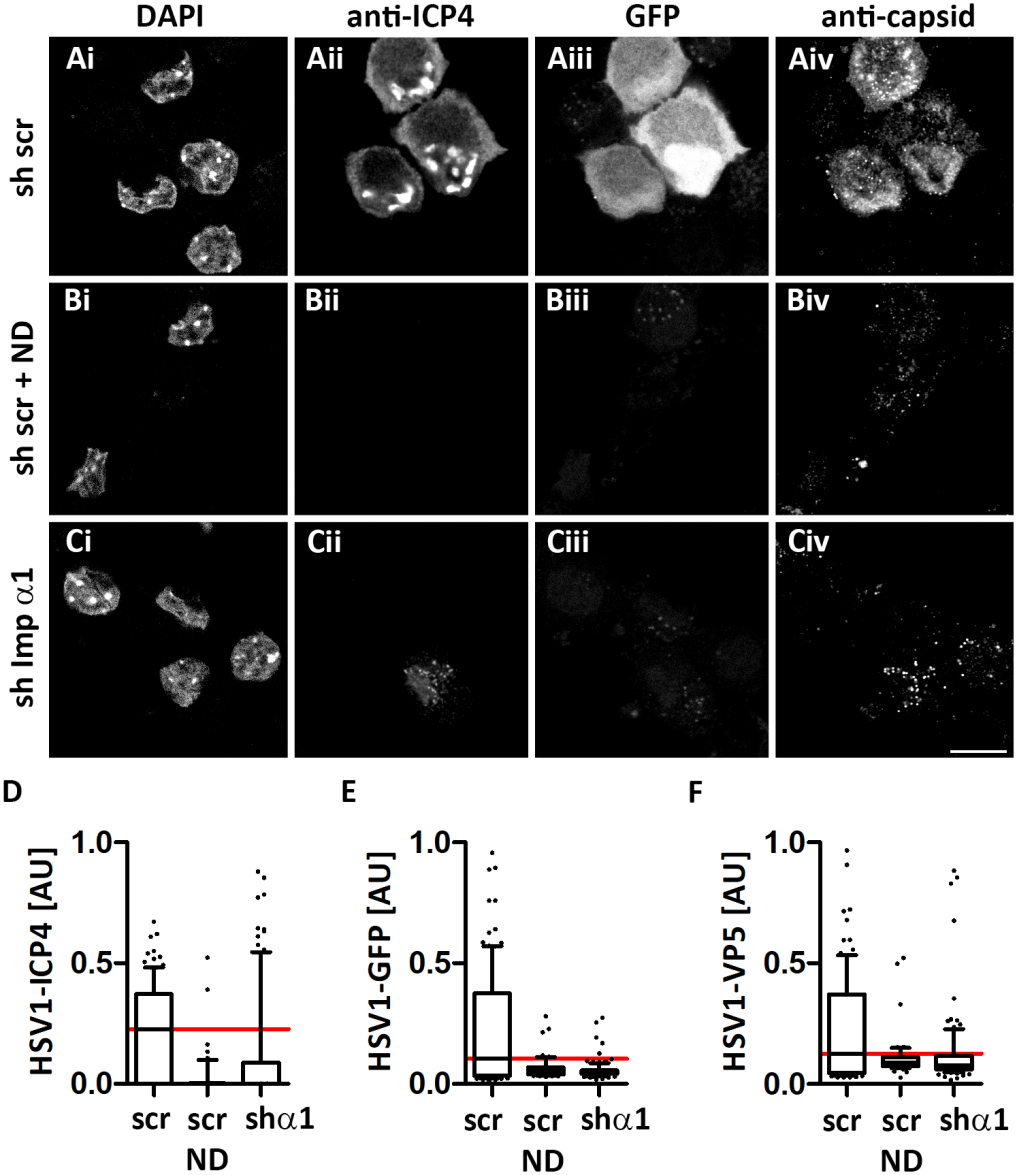
Importin α1 is required for HSV-1 gene expression in neurons. Primary cells from dorsal root ganglia cultured on cover slips were transduced with a scrambled shRNA (A, B) or an shRNA targeting importin α1 (C). At 7 dpt, the neurons were infected with HSV1(17^+^)Lox-GFP (5 x 10^6^ pfu/mL) in the absence (A, C) or presence of 10 μM nocodazole (B). At 4 hpi, the cells were fixed and permeabilized with PHEMO-fix, stained with DAPI (i), labelled with antibodies directed against ICP4 (ii) or capsids (iv), and analyzed by confocal fluorescence microscopy. GFP was detected by its intrinsic fluorescence (iii). Scale bar: 10 μm. The intra-nuclear ICP4 (D), GFP (E) and VP5 (F) signals were quantified with a CellProfiler pipeline using 109 (sh scr), 51 (sh scr + ND), and 106 (sh Imp α1) neurons and are shown as box plots with medians and whiskers representing the 10 to 90% percentile.

To further assess later stages of the HSV-1 life cycle, we infected neurons with HSV1(17^+^)Lox-GFP for 4 h, and labeled them for DNA, ICP4 and capsids. Neurons expressing the scrambled shRNA were well infected as indicated by nuclear targeting of ICP4 (Fig 8Aii), expression of the reporter GFP (Fig 8Aiii), and nuclear and cytoplasmic progeny capsids (Fig 8Aiv). In contrast, there was little expression of ICP4 (Fig 8Cii) or of GFP (Fig 8Ciii), and only incoming capsids were detected at the nuclear rims (Fig 8Civ) after importin α1 expression had been silenced. When the neurons had been infected in the presence of nocodazole, the incoming capsids were rather distributed over the cytoplasm than at the nuclear rims (Fig 8B). A quantitation of these signals in more than 50 neurons revealed that silencing importin α1 had reduced ICP4 (Fig 8D) and GFP (Fig 8E) expression and also the formation of nuclear capsid assembly compartments (Fig 8F) almost as efficiently as the nocodazole treatment. These experiments show that in primary neurons nuclear ICP4 expression, HSV1-mediated GFP expression, VP22 expression, and the formation of nuclear capsid assembly compartments depended on importin α1.

## Discussion

The herpesvirus life cycle depends on many nuclear functions, and we therefore tested the relevance of nuclear transport factors during infection. Our RNAi screen targeting 17 host transport factors demonstrated that importin β1, importin α1, importin α6, and transportin 1 were important for efficient HSV1-mediated GFP reporter expression in HeLa cells. A reduction in HSV-1 gene expression upon silencing importin β might have been expected as an NLS in the capsid associated tegument protein pUL36 and importin β are required to dock incoming HSV-1 capsids to the NPCs, and to inject their genomes into the nucleoplasm [23,25–29]. Subsequent experiments showed that importin α1 and importin α3 were required for efficient nuclear import of crucial HSV-1 proteins and infection of fibroblasts (c.f. S2 Table for a summary). Furthermore, silencing importin α1 expression in neurons abolished the formation of nuclear replication and capsid assembly compartments. While the lack of importin α1 or importin α3 delayed but did not prevent replication in fibroblasts, HSV-1 infection was dependent on importin α1 in differentiated neurons. Our data suggest that in neurons, HSV-1 infection requires specifically importin α1 and importin α3, whereas in dividing cell lines the lack of these importin α isoforms could be partially compensated, possibly by another importin α isoform. In view of their high sequence conservation ([1–4]; reviewed in [5]), our study revealed a remarkable specificity for distinct importin α isoforms required during HSV-1 infection.

While we focused here on importin α, future studies have to address the role of the other nuclear transport factors which were potential hits of our RNAi screen. Transportin 1 is another nuclear import factor that interacts with proline-tyrosine NLSs, such as e.g. those in hnRNP1 [88], which differ from the NLSs of the importin αs. Interestingly, we identified importin 9, 8, 11, 13, and transportin 3 as potential HSV-1 restriction factors. Importin 9 mediates the nuclear import of actin that is required for maximal host transcriptional activity [89], but apparently restricted HSV1-mediated GFP expression. Similarly, transportin 3 mediates nuclear import of splicing factors and has been implicated in HIV replication [90], but also seemed to restrict HSV-1. A depletion of importin 8 interferes with miRNA-guided gene silencing and RNA metabolism [91]. Importin 11 mediates nuclear import of E2 ubiquitin-conjugating enzymes [92,93], and importin 13 the bidirectional nuclear transport of the E2 SUMO-conjugating enzyme Ubc9 that catalyzes post-translational modifications important for intrinsic antiviral resistance [94,95]. Considering the diverse regulatory functions of transcription, miRNA, sumoylation and ubiquitination, it will be a challenge to dissect potential specific contributions of importin 9, transportin 3, importin 8, importin 11, and importin 13 to HSV-1 replication.

### Importins and HSV-1 infection of fibroblasts

The production of infectious cell-associated and extracellular virions was delayed and nuclear targeting of ICP4, ICP0, ICP8 and the DNA polymerase subunit pUL42 impaired in the MEF cells lacking importin α1 or importin α3. In contrast, nuclear targeting of incoming capsids as well as nuclear import of VP16 and the HSV-1 genomes seemed not to be affected. Although we could not test this directly since we lack sufficiently powerful antibodies, we suppose that HCF-1 had co-imported VP16 into the nucleus, and together with other nuclear host transcription factors such as Oct-1, SP1 and GABP initiated immediate-early transcription. The nuclear functions of HCF-1 are essential for cell viability, as regulatory processes controlled by this critical transcription factor do not operate properly, when HCF-1 is sequestered experimentally to the cytosol [96]. Consistent with this assumption, we detected similar expression levels of the immediate-early protein ICP4 by immunoblot in the different MEF lines. However, the nuclear import of ICP4 and another immediate-early protein ICP0 was severely impaired without importin α1 or importin α3. Based on the coordinated interdependent and temporally regulated HSV-1 expression program reported in other systems [30,31,33], we expected that reducing the nuclear amounts of ICP4 and ICP0 would delay subsequent steps of the HSV-1 life cycle. Yet, expression of the early and late proteins ICP8, VP16, and VP22 was not or only moderately reduced in MEFs lacking importin α1 or importin α3, and even increased in the absence of importin α4.

Although HSV-1 gene expression seemed rather unperturbed, the nuclear import of the ssDNA binding protein ICP8 and the DNA polymerase processivity factor pUL42 were reduced in the absence of importin α1 or importin α3. The two DNA polymerase subunits pUL30 and pUL42 of HSV-1 rely on several mechanisms for nuclear import, and can be imported individually or as a holoenzyme (reviewed in [56]). HSV1-pUL30 comprises a non-canonical and a classical bipartite NLS, and binds to importin α5, but other importin α isoforms have not been tested [97–99]. A bipartite NLS in HSV1-pUL42 has been shown to bind to importin α7 and to some extent to importin α1 but actually not to importin α3 [100]; nevertheless its nuclear import was reduced in the absence of importin α1 or importin α3. pUL30 and pUL42 with mutated NLSs are still efficiently imported and targeted to the DNA replication compartments when co-expressed with the wild-type version of the other, but the holoenzyme is retained in the cytosol when the NLSs on both subunits are mutated [100]. Thus, it is possible that the lowered amounts of nuclear ICP8 were sufficient to sustain some DNA replication by a nuclear pUL30 despite reduced amounts of its accessory factor pUL42. Importin α1 was a hit in our targeted RNAi screen for HSV1-mediated GFP expression; possibly because the nuclear HSV1 DNA replication had been reduced. Furthermore, the nuclear import of one of the host factors NF-ΚB, CREB/ATF, AP-1, or SP1 that bind to the major immediate-early promotor of murine cytomegalovirus controlling GFP expression in our reporter virus might have been impaired [101,102].

Although immediate-early, early and late HSV-1 proteins had been synthesized, the electron microscopy analysis shows that the assembly of nuclear capsids, and thus the overall amount of capsids was significantly reduced in the absence of importin α1. Furthermore, the targeting of the HSV-1 pUL31/pUL34 nuclear export complex to the inner nuclear membrane (reviewed in [103,104]) might have been impaired, leading to the reduced nuclear egress of progeny capsids, and the reduced amount of cytoplasmic capsids. Consistent with an overall reduced nuclear targeting of important HSV-1 proteins, a reduced formation of nuclear capsids, and a reduction in nuclear egress, the production of infectious HSV-1 virions was delayed but not prevented in MEF-Impα1^-/-^, and to some extent also in MEF-Impα3^-/-^. The specific requirement for importin α3 over importin α4 is remarkable, considering that their amino acid sequences are to 86% identical and to 92% conserved, and considering that importin α4 might even restrict certain steps of the HSV-1 replication cycle. It may nevertheless be possible that when one importin α is missing, the HSV-1 proteins could utilize another importin α homolog.

### Importins and HSV-1 infection of neurons

In the differentiated, post-mitotic neurons, HSV-1 infection depended even more on importin α1 and importin α3. When importin α1 expression had been reduced by RNAi, the amounts of ICP4, HSV-1 mediated GFP, VP22, as well as the formation of nuclear capsid assembly compartments were reduced, while nuclear targeting of incoming capsids was not inhibited irrespective of an inoculation via the somal plasma membrane or the axons. The distribution of importin α isoforms is highly regulated in different cell types and during development (reviewed in [3,5,6]). During neuronal differentiation, expression changes from being initially high in importin α1 and low in importin α3 and importin α5 to low in importin α1 and high in importin α3 and importin α5 [105]. The importin α repertoire of post-mitotic neurons might be more limited than that of MEFs, and therefore silencing the expression of importin α1 or importin α3 had a stronger impact on HSV-1 infection in neurons.

Having available the novel knock-out mice [73,106], MEF lines lacking specific importin α isoforms [2,12,73], and shRNA lentiviral vectors targeting specific importin α isoforms without influencing the expression of other importin α isoforms, we could validate antibodies specific for particular importin α isoforms or subfamilies. While importin α1 has been considered the general nuclear transport factor for cargoes with a classical NLS [2], we and others could generate knock-out mice for specific importin αs suggesting that their host functions could be compensated at least to some extent [73,106]. Our study contributes to elucidating the mode of importin α isoform specificity *in vivo* that is so far only understood for a limited number of cargoes (reviewed in [5]). Furthermore, not all binding reactions of a substrate to an importin α result in nuclear import of this substrate; for example, Oct-6 can bind to multiple importin-α isoforms, but while binding to importin α1 causes retention in the cytoplasm, binding to importin α5 results in nuclear import [11].

It will be interesting to determine, whether other alphaherpesviruses, betaherpesviruses, and gammaherpesviruses depend on the same importin α isoforms for viral protein import into the nucleus, capsid assembly, and capsid egress to the cytoplasm. Since the early years of the nuclear transport field, the interaction of viral proteins with import factors has been studied, and in several proteins of the herpesviruses and also other viruses replicating in the nucleus, NLS motifs recruiting specific import factors have been identified (for review see [5,56]). Interestingly, the polymerase subunit PB2 of avian influenza A virus strains, an RNA virus replicating in the nucleus, preferentially binds to importin α3, while mammalian adapted strains prefer importin α7, and this switch might be a virulence factor in avian-mammalian host adaptation [107]. Other viruses actually do not utilize but disarm specific importin α isoforms. The structural protein VP24 of Ebola virus and the polymerase of hepatitis B virus block the nuclear import of STAT1, and thus interferon signaling by competitive binding to importin α5 [108–110].

Although the exact intracellular concentration of different nuclear transport factors is hard to measure *in situ*, it will be interesting to determine to which extent the specific importin isoforms are expressed in epithelial cells, fibroblasts, neurons, and immune cells that are targeted by HSV-1 and other herpesviruses. In future work, it may be possible to reduce expression of all isoforms of one importin α subfamily in cell lines or in primary cells derived from tissues of these knock-out mice in order to reveal potentially redundant virus-host interactions. Further binding studies using recombinant HSV-1 proteins and limiting and competing amounts of different importins will dissect whether herpesvirus proteins comprise additional binding determinants that provide preferential specificity for importin α1 and importin α3 in addition to the already known NLSs. Finally, herpesviruses may also utilize NLSs of tegument proteins, e.g. the one in the N-terminal part of pUL36, or in capsid proteins exposed on the surface of the incoming capsids to recruit specific importin α isoforms and importin β for capsid targeting to the nuclear pores for genome release into the nucleoplasm.

## Materials and methods

### Cells

All cell lines were maintained as adherent cultures in a humidified incubator at 37°C and 5% CO_2_ and passaged twice per week. BHK 21 cells (ATCC CCL-10) and Vero-D6.1 expressing HSV1-gB (Helena Browne, University of Cambridge, personal communication; [78]) were maintained in minimum essential medium (MEM; Cytogen, Wetzlar, Germany) supplemented with 10% (v/v) FCS (PAA Laboratories GmbH, Cölbe, Germany; Life Technologies Gibco) and Vero cells (ATCC CCL-81) in MEM supplemented with 7.5% FCS. HeLaCNX cells [62], human embryonic kidney cells (HEK293T, ATCC CRL-11268; [111]) and mouse embryonic fibroblasts (MEFs) derived from wild type (MEF^wt^), MEF-Impα1^-/-^ from importin α1^-/-^, MEF-Impα3^-/-^ from importin α3^-/-^, and MEF-Impα4^-/-^ from importin α4^-/-^ [73] C57Bl/6 mice were cultured in Dulbecco’s modified Eagle’s medium (DMEM)-GlutaMAX-I (Life Technologies Gibco, Darmstadt, Germany) supplemented with 10% (v/v) FCS.

Cells from DRG of adult C57Bl/6JHanZtm mice were cultured using established protocols [83,112–114]. The mice strain C57Bl/6JHanZtm (not genetically modified) were bred and maintained without any perturbation. On the day of the experiment, they were taken up from the animal facility, within 3 hours sedated with CO_2_-inhalation prior to killing by cervical dislocation without any prior experimental perturbation, and the DRG from the cervical, thoracic and lumbar levels of 3 to 4 mice were dissected afterwards. Those DRG were pooled in 1x HBSS-complete buffer (Hank’s balanced salt solution, pH 7.4 with 5 mM HEPES and 10 mM D-Glucose), incubated with 20 mg/mL papain (Sigma-Aldrich; in 0.4 mg/mL L-Cysteine, 0.5 mM EDTA, 1.5 mM CaCl_2_xH_2_O, pH 7.4) for 20 min at 37°C, with 10 mg/mL collagenase IV (Invitrogen) and 12 mg/mL dispase II (Sigma-Aldrich) for another 20 min at 37°C, and then triturated using Pasteur pipettes with narrowed ends. The cells were sedimented through 20% (v/v) Percoll (Sigma-Aldrich) cushions in CO_2_-independent medium (Life Technologies Gibco, Carlsbad, CA, USA) containing 10 mM D-glucose, 5 mM HEPES, 10% FCS, 100 U/mL penicillin and 0.1 mg/mL streptomycin, suspended in Ham’s F-12 nutrient mix medium with 10% FCS, 50 ng/mL 2.5S nerve growth factor (Promega Corporation, Fitchburg, WI, US), 100 U/mL penicillin and 0.1 mg/mL streptomycin, and seeded onto cover slips of 20 mm diameter in 24-well plates or into microfluidic devices (SND 450, Xona Microfluidics, LLC, Temecula, CA, USA) attached to 24 x 32 mm cover slips. The cover slips had been pre-coated with 0.01% (w/v) poly-L-lysine (Sigma-Aldrich) and 7 ng/μl murine laminin (Invitrogen). The cells were cultured at 37°C and 5% CO_2_ in a humidified incubator, and the media were replaced twice a week. The mitosis inhibitor 1-β-D-arabinofuranosylcytosine (Sigma-Aldrich) was added at 1 to 2 div to a final concentration of 2 μM to suppress proliferation of dividing, non-neuronal cells, but removed at 4 div prior to HSV-1 infection.

### Viruses

We used HSV1(17^+^)Lox, HSV1(17^+^)Lox-_pMCMV_GFP, or HSV1-GFP for short, which expresses soluble GFP under the control of the major immediate-early promoter of murine cytomegalovirus [62], HSV1(17^+^)Lox-CheVP26, in which monomeric Cherry has been fused to the N-terminus of VP26 [76], HSV1(17^+^)Lox-CheVP26-UL37GFP [76], and HSV1(17^+^)Lox-ΔgB lacking the UL27 gene that encodes the essential glycoprotein gB [77]. Virus titers were assessed by plaque assays [115], or for HSV1(17^+^)Lox-ΔgB estimated by comparing an immunoblot analysis of extracellular viral particles to HSV1(17^+^)Lox-_pMCMV_GFP expressing gB and used in parallel. For infection experiments, extracellular virus sedimented from the medium of infected BHK 21 cells was used [18,115].

### Plaque assays

The stocks of the different HSV-1 strains used for infection as well as the MEF-associated virus and the virus released from infected MEFs were titrated on Vero cells. At 4, 8, 12, 16 and 20 hpi, the supernatants of infected MEF were collected and cleared by low-speed sedimentation, and the cells were scraped into 1 mL/well MNT buffer (30 mM MES, 100 mM KCl, 20 mM Tris, pH 7.4) and subjected to 3 cycles of freeze-thawing. Vero cells were cultured to just confluency in 6-well dishes, and incubated for 1 h at room temperature on a rocking platform with 10-fold serial dilutions of the different virus suspensions in CO_2_-independent medium (Life Technologies Gibco) with 0.1% [w/v] cell culture grade bovine serum albumin (PAA Laboratories GmbH). The inoculum was removed and 2 mL/well growth medium containing 20 μg/mL pooled human IgG (Sigma-Aldrich) was added. The cells were incubated for 3 d, fixed in absolute methanol, and stained with 0.1% [w/v] crystal violet and 2% [v/v] ethanol in H_2_O.

### Antibodies and other reagents

To stain DNA, we used 4’,6-diamidino-2-phenylindole (DAPI; Roth) or TO-PRO-3-iodide (Life Technologies) at final concentrations of 50 μg/mL or 1 to 2 μM, respectively. We used rabbit polyclonal antibodies (pAbs) raised against human importin α1 (#70160, Abcam), human importin α3 (Enno 31; Pineda Antikörper Service, Berlin, Germany), human importin α4 (Enno 32; Pineda Antikörper Service), human importin α5/α6/α7 (MDC 220; [2]), HSV1-VP16 (#631209, BD Biosciences), HSV-1 tegumented capsids (Remus, bleed V; [23]), or nuclear HSV-1 capsids. To generate a polyclonal serum directed against HSV-1 capsids (SY4563, anti-capsid), rabbits were immunized with purified nuclear capsids (Kaneka Eurogentec S.A., Seraing, Belgium). Mouse monoclonal antibodies (mAb) were directed against α-tubulin (DM1A, Sigma-Aldrich), nuclear pore complexes (mAb 414, Abcam), actin (mAb 1501, Millipore), β-III-tubulin (mAb 5564, Millipore), p150^Glued^ (#610474, BD Biosciences), HSV1-ICP0 (mAb 11060, sc-53070, Santa Cruz Biotechnology), HSV1-ICP4 (mAb 10F1, ab6514, Abcam), HSV1-ICP8 (mAb 11E2, ab20194, Abcam), or HSV1-pUL42 (ab19311, Abcam). Secondary antibodies for immunoblotting were conjugated to fluorescent infrared dyes (anti-rabbit IgG-IRDye 800CW, anti-mouse IgG-IRDye 680RD, LI-COR Biosciences), and for immunofluorescence microscopy to Cy3 (goat-anti-rabbit IgG; Dianova), Cy5 (goat anti-mouse IgG; Dianova), Alexa Fluor488 (A488; goat anti-rabbit IgG; goat-anti-mouse IgG, Invitrogen) or fluorescein isothiocyanate (FITC; goat anti-rabbit IgG; Dianova). All secondary antibodies were highly pre-adsorbed to eliminate cross-reactivity to other species than the intended one.

### Lentiviral vectors expressing shRNAs

To silence importin α1, importin α3, or importin α4 by short hairpin RNAs (shRNAs; Sigma Mission library; S3 Table) or to express a non-mammalian shRNA control (SHC002, Sigma Mission library), we used lentiviral transduction. HEK 293T cells were transfected with 5 μg pRSVRev, 2 μg pMD2.g (Addgene Inc., Cambridge, MA, USA, Cat. No. 12259), 10 μg pCDNA3.GP.CCCC, and 10 μg transfer plasmid per 10 cm dish as described previously ([116]; plasmids provided by Axel Schambach). The supernatants were harvested at 36 and 48 h, and sedimented in a SW32.Ti rotor at 24,000 rpm for 90 min at 4°C (Beckman Coulter). The re-suspended lentiviral particles were snap frozen in liquid N_2_ and stored in single-use aliquots at -80°C. Cell culture supernatants and concentrated lentiviral stocks were titrated using a p24 ELISA [117]. MEF^wt^ were transduced with lentiviral particles at 4 to 12 μg/mL p24 and at 1 dpt, selection with puromycin at 2.5 μg/mL was started. DRG cells were transferred after 1 day *in vitro* to neuronal growth media containing lentiviral particles at 4 to 12 μg/mL p24 but no AraC. After 2 dpt, the media were replaced by F12-complete with 2 μM AraC and 5 μg/mL puromycin to select for transduced cells.

### RNAi screen

Small interfering RNAs (siRNAs) against human transport factors as well as scr siRNAs were from QIAGEN (c.f. S1 Table; Hilden, Germany) and the GFP silencer siRNAs from Ambion (AM4626; Darmstadt, Germany). 3,500 to 4,000 HeLaCNX cells per well of 96-well plates were reverse transfected with 50 nM of siRNA using Lipofectamine 2000 (Invitrogen, Life Technologies). After 3 days, cells were left untreated or pre-treated with 50 μM nocodazole for 1 h and infected with 4 x 10^6^ PFU/mL of HSV1(17^+^)Lox-_pMCMV_GFP for 12 h in the absence or presence of nocodazole. Cells were fixed with 3.4% paraformaldehyde (PFA), permeabilized with 0.1% Triton-X-100 and stained with DAPI. DAPI and GFP fluorescence were measured using a fluorescence plate reader (BioTek Synergy 2, Bad Friedrichshall, Germany) and the GFP background signal of the mock infected cells was subtracted. To allow comparison of different experiments, the median values of cells transfected with scr siRNAs of each experiment were set as 100% and GFP/well and DAPI/well values were calculated. To reduce the impact of potential off-target effects introduced by miRNAs binding the siRNA seed region, the results were corrected using a dataset of seed region phenotypes [66]. The seed regions of siRNAs classified by Franceschini et al. (2014) to result in off-target effects were compiled, and the mean of significantly altered seed region phenotypes were determined using a threshold of p< = 0.05 after Bonferroni correction [118]. Franceschini et al. (2014) propose an additive model with the seed phenotype contributing with a factor of 0.6 to the overall gene expression results. This adjusted seed phenotype was subtracted from the gene expression results, and the medians of GFP/well, GFP^corr^/well or DAPI/well respectively were determined (c.f. S1 Table, GFP, GFP^corr^, DAPI). To normalize for potential effects of RNAi on cell density, GFP^corr^/DAPI ratios were determined for each well, and the median from the single values was calculated (c.f. S1 Table, GFP^corr^/DAPI).

### HSV-1 infection

For immunofluorescence microscopy, immunoblot analysis and viral growth curves, MEFs were seeded onto coverslips in 24-well plates at densities of 1 x 10^5^ cells/well or into 6-well dishes at 2.5 x 10^5^ cells/well, and on the next day pre-cooled and inoculated with HSV-1 in CO_2_-independent medium with 0.1% (w/v) cell culture grade bovine serum albumin (BSA; PAA Laboratories GmbH). MEFs were inoculated for 1 h on ice for nuclear targeting assays, for 0.5 h on ice for measuring nuclear import of viral genomes and VP16, and for 2 h at RT for measuring viral gene expression by immunoblot and measuring nuclear import of viral proteins by confocal fluorescence microscopy. DRG cells were inoculated for 0.5 h at RT. After washing off the unbound virions, the cells were shifted to growth medium at 37°C for the indicated times. We used 5 x 10^7^ pfu/mL (MOI of 100) of HSV1(17^+^)Lox-CheVP26 to analyze nuclear targeting of incoming HSV-1 capsids, 1 x 10^8^ pfu/mL (MOI 200) of HSV1(17^+^)Lox-_pMCMV_GFP or of HSV1(17^+^)Lox–ΔgB to study the subcellular localization of incoming VP16, 1 x 10^8^ pfu/mL (MOI of 200) of HSV1(17^+^)Lox-CheVP26-UL37GFP to examine the nuclear import of incoming viral genomes, 0.5 to 1.25 x 10^6^ pfu/mL (MOI 2 to 5) of HSV1(17^+^)Lox-CheVP26 to examine the synthesis of structural HSV-1 proteins by immunoblot, 0.5 to 1.25 x 10^6^ pfu/mL (MOI 2 to 5) of HSV1(17^+^)Lox-CheVP26 to determine the subcellular localization of the immediate-early proteins ICP4 and ICP0, the early proteins ICP8, and the late structural protein CheVP26. For the virus growth curves, the different MEF lines were infected with 1.3 x 10^6^ pfu/mL (MOI 5) of HSV1(17^+^)Lox at a reduced level of 1% [v/v] FCS. Primary cells derived from the DRGs were infected with 2.5 x 10^7^ pfu/mL for nuclear capsid targeting, with 5 x 10^6^ pfu/mL for gene expression upon infection from the somal plasma membrane, or with 1.3 x 10^8^ pfu/mL for nuclear capsid targeting upon infection from the axonal compartment in microfluidic chambers. In those experiments analyzing the subcellular localization of incoming HSV-1 capsids, incoming VP16 or incoming viral genomes, 0.5 mM cycloheximide (Sigma-Aldrich) was added to prevent synthesis of new viral proteins [18]. When nocodazole (25 or 50 μM for MEFs, 10 μM for neurons; Sigma-Aldrich) was used to depolymerize microtubules, cells were pretreated for 1 h at 37°C, and the drug was present during all further steps.

### Immunoblotting

Cells were lysed in hot sample buffer (1% [w/v] SDS, 50 mM Tris-HCl, pH 6.8, 1% [v/v] β-mercaptoethanol, 5% [v/v] glycerol bromophenol blue) containing a protease inhibitor cocktail (cOmplete Roche, #11873580001), and the DNA was sheared using 20-gauge needles. The lysates were loaded onto linear 5 to 12% gradient or 10% SDS gels, and proteins were transferred to nitrocellulose membranes. Membranes were incubated with a blocking solution of 5% [w/v] low-fat milk in PBS followed by incubation with primary antibodies in blocking solution, washed with PBS containing 0.1% [w/v] Tween-20 and 0.5% milk, incubated with secondary antibodies in blocking solution, washed and scanned (Odyssey Infrared Imaging System, LI-COR Biosciences, NE, USA). The band areas and mean intensities were measured using a rectangular selection tool to calculate the integrated intensity (ImageJ version 1.50e, NIH, USA). The background was subtracted, the integrated intensities were normalized to untreated MEF^wt^, and the ratios of the respective viral protein to actin used as loading control were calculated.

### Immunofluorescence microscopy

Infected cells were either simultaneously fixed and permeabilized with PHEMO-fix (68 mM PIPES, 25 mM HEPES, 15 mM EGTA, 3 mM MgCl_2_, 10% [v/v] DMSO, 3.7% [w/v] PFA, 0.05% [v/v] glutaraldehyde, 0.5% [v/v] Triton X-100, pH 6.9) for 10 min at 37°C and washed twice with PHEMO buffer (68 mM PIPES, 25 mM HEPES, 15 mM EGTA, 3 mM MgCl_2_, 10% [v/v] DMSO, pH 6.9), or fixed with 3% [w/v] PFA in PBS for 20 min at room temperature as described before [18,19]. Fixed cells were treated with 50 mM NH_4_Cl/PBS for 10 min, and permeabilized with 0.1% Triton X-100/PBS for 5 min in the case of PFA fixation. The HSV1-Fc receptor [119] and other unspecific antibody binding were blocked with 0.5% (w/v) BSA and 10% (v/v) serum from HSV1-negative volunteers. After the immunolabelling, the samples were embedded in Mowiol containing 10% [w/v] 1,4-diazabicyclo[2.2.2]octane, and imaged with plan-apochromatic 63x oil-immersion objectives with a numerical aperture of 1.4 with a confocal fluorescence microscope (LSM 510 Meta; Carl Zeiss Microscopy, Jena, Germany; TCS SP6, LEICA Microsystems, Wetzlar, Germay). Contrast and brightness were adjusted identically across each set of images (Adobe Photoshop version 6.0 or version CS4). Figures were assembled using Adobe Illustrator CC (version 20.1.0).

To quantify the nuclear accumulation of ICP4, ICP0, ICP8, pUL42, GFP or capsid proteins, we developed a pipeline using the CellProfiler software ([120]; http://cellprofiler.org/; BI-2013-070, version 2.1.1, NIH, USA) that segmented the nuclei based on DAPI fluorescence and size, and then determined the mean fluorescence intensity of the labeling for the above mentioned proteins. To measure the number of capsids at the nuclear rim of neurons, nuclear corridors around the outer rim of the segmented nuclei were defined by both expanding and shrinking the nuclear area by several pixels. Then the number of capsids localized within that area was counted. Thresholds for the recognition of the capsid signal were based on the typical signal intensity and size of capsids and considering the background intensity of the anti-capsid antibody in uninfected neurons. For each protein, the average grey values per nuclei were calculated to compile box and whisker plots. The p values were determined with a Kruskal-Wallis test followed by Dunn’s multiple comparison testing (software Prism, version 6; Graphpad, San Diego, CA, USA).

### Fluorescent *in situ* hybridization (FISH)

To analyze the subcellular distribution of incoming HSV-1 genomes, cells were infected as described above and fixed with a mixture of 95% ethanol and 5% acetic acid, and processed for fluorescent *in situ* hybridization (FISH). HSV-1 probe synthesis and hybridization were performed as described previously [121,122] using a HSV1(17^+^)Lox-ΔUL36 genome cloned into a bacterial artificial chromosome [123] to generate a Cy3-labelled DNA probe. For detection of incoming HSV-1 genomes, the DNA probe was used at 20 μg per coverslip, and the samples were analyzed by confocal fluorescence microscopy.

### Electron microscopy

MEF^wt^ or MEF-Impα1^-/-^ seeded on glass cover slips were infected with HSV1(17^+^)Lox with an MOI of 10 pfu/cell at 2.5 x 10^6^ pfu/mL. The cells were fixed at 12 hpi with 2% glutaraldehyde and 2.5% formaldehyde in cacodylate buffer [130 mM (CH_3_)_2_AsO_2_H, pH 7.4, 2 mM CaCl_2_, 10 mM MgCl_2_] for 1 h at room temperature. Cells were contrasted with 1% (w/v) OsO_4_ in cacodylate buffer (165 mM (CH_3_)_2_AsO_2_H, pH 7.4, 1.5% (w/v) K_3_[Fe(CH)_6_]) followed by 0.5% (w/v) uranyl acetate in 50% (v/v) ethanol overnight. The cells were embedded in Epon plasticServa, Heidelberg, Germany) and 50 nm ultrathin sections were cut parallel to the substrate. Images were acquired with a Morgani transmission electron microscope (FEI, Eindhoven, The Netherlands) at 80 kV. Viral structures were counted and sectioned nuclear and cytoplasmic areas were measured using Fiji software (fiji.sc).

### Ethic statement

Human sera of exclusively adult, healthy, HSV-1 seronegative volunteers were obtained after written informed consent by the blood donors. Permission was granted by the Institution Review Board (Hannover Medical School; Approval Number 893). According to the German Animal Welfare Law §4, killing of animals needs no approval, if the removal of organs serves scientific purposes, and if the mice had not undergone experimental treatment before. The animal care and sacrifices were performed in strict accordance with the German regulations of the Society for Laboratory Animal Science (GV-SOLAS), the European Health Law of the Federation of Laboratory Animal Science Association (FELASA) and the German Animal Welfare Law. This study here does not contain animal experiments that require pre-approval, and the total number of killed mice was reported at the end of each year to the animal welfare deputy of Hannover Medical School. This information was registered annually as the number of animals killed according to §4 of the German Animal Welfare Law and the number of killed mice was registered with the animal welfare application number 2012/20 at the local state authority (LAVES; *Niedersächsisches Landesamt fuer Verbraucherschutz und Lebensmittelsicherheit*, Oldenburg, Germany).

## Supporting information

S1 Fig Importin α expression in human and murine cells. (A) MEF^wt^, MEF-Imp α1^-/-^, MEF-Imp α3^-/-^ or MEF-Imp α4^-/-^ were seeded at 2.5 x 10^6^ cells per 10 cm dish for 16 h, lysed and analyzed by immunoblot using antibodies against p150^Glued^, importin α1, α3, α4, α5/α7 or actin. (B) MEF^wt^ were transduced for 7 days with scr shRNA or with shRNAs targeting murine importin α1, α3 or α4. Cell lysates were analyzed by immunoblot using antibodies against p150^Glued^, importin α1, α3, α4 or α5/α7.
(TIF)

S2 Fig Importin α1, α3, or α4 are not required for nuclear import of incoming HSV-1 genomes or HSV1-VP16. A-E: MEF^wt^ (A, B), MEF-Impα1^-/-^ (C), MEF-Impα3^-/-^ (D) or MEF-Impα4^-/-^ (E) were inoculated with HSV1(17^+^)Lox-CheVP26-UL37GFP (1 x 10^8^ pfu/mL, MOI of 200) or mock treated (F; MEF^wt^ only) in the presence of cycloheximide. At 3 hpi, the cells were fixed and denatured with a mixture of 95% ethanol and 5% acetic acid, hybridized with BAC-derived HSV1(17^+^)Lox-Cy3-DNA (iv), and analyzed by confocal microscopy. The boxed area in ii is presented at higher magnification in iii–v. The blue lines (iv) indicate position of the nuclei as determined by DIC (i). Scale bar, 20 μm. F-K: MEF^wt^ (F, G & K), MEF-Impα1^-/-^ (H), MEF-Impα3^-/-^ (I) or MEF-Impα4^-/-^ (J) were inoculated with HSV1(17^+^)Lox-GFP (F-J; 1 x 10^8^ pfu/mL, MOI of 200) or with HSV1(17^+^)Lox-ΔgB (K) with a comparable number of viral particles in the presence of cycloheximide (F, H-K) or of cycloheximide and nocodazole (G). The cells were fixed and permeabilized with PHEMO-fix at 4 hpi, labeled with antibodies against VP16 (i), stained with TO-PRO-3 (ii; blue line in i), and analyzed by confocal microscopy.
(TIF)

S3 Fig Microtubule and nuclear pore organization unchanged in MEFs. (A) Confocal microscopy of MEF^wt^ (Ai), MEF-Impα1^-/-^ (Aii), MEF-Impα3^-/-^ (Aiii), and MEF-Impα4^-/-^ (Aiv) mock treated in the presence of cycloheximide for 4 h, fixed and permeabilized with PHEMO-fix and labeled with antibodies against α tubulin. (B) Confocal microscopy of MEF^wt^ (Bi), MEF-Impα1^-/-^ (Bii), MEF-Impα3^-/-^ (Biii), and MEF-Impα4^-/-^ (Biv) inoculated with HSV1(17^+^)Lox-CheVP26 (5 x 10^7^ pfu/mL; MOI of 100) for 5 h in the presence of cycloheximide, fixed and permeabilized with PHEMO-fix and labeled with antibodies against NPC. Scale bar: 10 μm.
(TIF)

S4 Fig Importin α1 facilitates and importin α4 restricts efficient HSV-1 protein expression. (A) MEF^wt^, MEF-Imp α1^-/-^, MEF-Imp α3^-/-^, or MEF-Imp α4^-/-^ were mock infected or infected for 6 h with HSV1(17^+^)Lox-CheVP26 (0.5 to 1.25 x 10^6^ pfu/mL, MOI of 2 to 5 in the absence or presence of nocodazole (ND). To estimate HSV-1 expression levels upon different perturbations, 25%, 50% or 100% of a MEF^wt^ lysates were loaded for comparison. The lysates were analyzed by immunoblot using antibodies against ICP4, ICP8, several HSV-1 structural proteins including VP16 and VP22 (pAb Remus V), or actin as a loading control. The upper part of the membrane was first incubated with anti-ICP8 (130 kDa, 2^nd^ row) and then re-probed with anti-ICP4 (175 kDa; first row).
(TIF)

S5 Fig Importin α1 and α3 are required for nuclear localization of HSV-1 immediate-early and early proteins. MEF^wt^ (A, F, K), nocodazole treated MEF^wt^ (wt + ND; B, G, L), MEF-Impα1^-/-^ (C, H. M), MEF-Impα3^-/-^ (D, I, N), or MEF-Impα4^-/-^ (E, J, O) were infected with HSV1(17^+^)Lox-CheVP26 (0.5 to 1.25 x 10^6^ pfu/mL, MOI of 2 to 5), fixed at different times post infection with 3% PFA, permeabilized with TX-100, and labeled for ICP0 (A-E; 4 hpi), ICP8 (F-J; 6 hpi) or pUL42 (K-O; 8 hpi), and analyzed by confocal fluorescence microscopy. Scale bar 20 μm.
(TIF)

S6 Fig Importin α1 and α3 are required for the nuclear localization of HSV-1 immediate-early and early proteins. MEF^wt^ transduced with scr shRNA (A, B, F, G) or shRNAs targeting importin α1 (C, H), α3 (D, I) or α4 (E, J) were infected with HSV1(17^+^)Lox-CheVP26 (0.5 to 1.25 x 10^6^ pfu/mL, MOI of 2 to 5) in the absence (A, C-E, F, H-J) or presence of nocodazole (B, G). At 4 (A-E) or 6 (F-J) hpi, cells were fixed with 3% PFA, permeabilized with TX-100, labeled with antibodies directed against ICP4 (A-E) or ICP8 (F-J), and analyzed by confocal fluorescence microscopy.
(TIF)

S1 Table Specific nuclear transport factors are required for HSV-1 early gene expression. HeLaCNX cells were mock-treated or transfected with 50 nM of siRNA directed against different host transport factors in quadruplicate in 2 to 12 independent experiments (# of wells = 4 times # of exp.). After 3 days cells were left untreated or pre-treated with 50 μM nocodazole for 1 h and infected with 4 x 10^6^ PFU/mL of HSV1(17^+^)Lox-GFP for 12 h in the absence or presence of nocodazole. Cells were fixed, permeabilized, and stained with DAPI. GFP and DAPI fluorescence were measured using a fluorescence plate reader, and normalized to uninfected or DMSO treated, infected cells to express the data of different experiments as percentages (%). To reduce the impact of potential off-target effects introduced by miRNAs binding the siRNA seed region, the results were corrected using a dataset of seed region phenotypes. The seed regions of siRNAs classified by Franceschini et al. (2014) to result in off-target effects were compiled, and the mean of significantly altered seed region phenotypes were determined using a threshold of p< = 0.05 after Bonferroni correction (http://www.bioconductor.org/packages/release/bioc/manuals/scsR/man/scsR.pdf, page 26). To normalize for potential effects of RNAi on cell density, the HSV1-mediated GFP expression / cell density coefficients were calculated from the respective individual measurements: GFP—median before Bonferroni correction; GFP^corr^—median after Bonferroni correction; GFP^corr^/DAPI—median of individual GFP^corr^/DAPI ratios. The degree of inhibition of different siRNA were then ranked; first within one transport factor and then among all transport factors investigated (av x in %), and then also expressed in absolute numbers (relative rank).
(DOCX)

S2 Table HSV-1 infection experiments with MEFs. Summary of the results of the different experiments assessing different stages of the HSV-1 replication cycle in MEF-Impα1^-/-^, MEF-Impα3^-/-^, or MEF-Impα4^-/-^ lines in comparison to the MEF^wt^ control cell line, as well as in MEF^wt^ or DRG neurons transduced with shRNAs targeting importin α1, α3 or α4 in comparison to scr-transduced MEF^wt^ or neurons.
(DOCX)

S3 Table List of shRNA sequences tested and validated for silencing importin α. SIGMA TRCN numbers and sequences of the shRNAs used in this study and effect on target as assessed by immuno-blotting. Sequences used for infection assays in MEFs or DRG cells are indicated by x.
(DOCX)
