# Supplementary figures and images for: Importin α1 is required for nuclear import of herpes simplex virus proteins and capsid assembly in fibroblasts and neurons
Source: PLoS Pathog. 2018 Jan 5;14(1):e1006823. doi: 10.1371/journal.ppat.1006823 (PMC5773220; doi:10.1371/journal.ppat.1006823)

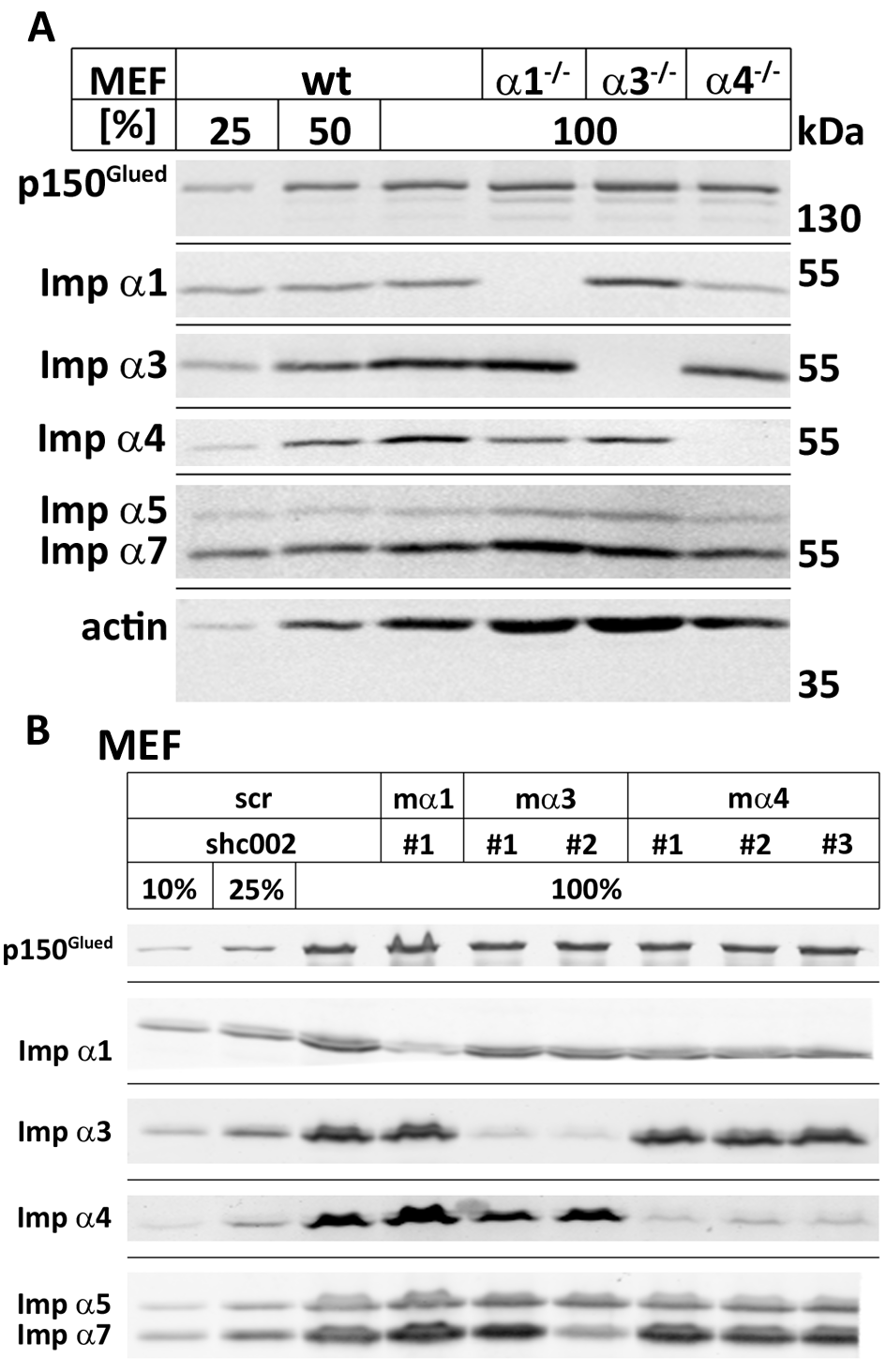

Supplement: S1 Fig — (A) MEFwt, MEF-Imp α1-/-, MEF-Imp α3-/- or MEF-Imp α4-/- were seeded at 2.5 x 10^6^ cells per 10 cm dish for 16 h, lysed and analyzed by immunoblot using antibodies against p150Glued, importin α1, α3, α4, α5/α7 or actin. (B) MEFwt were transduced for 7 days with scr shRNA or with shRNAs targeting murine importin α1, α3 or α4. Cell lysates were analyzed by immunoblot using antibodies against p150Glued, importin α1, α3, α4 or α5/α7. (TIF) [file ppat.1006823.s001.tif]

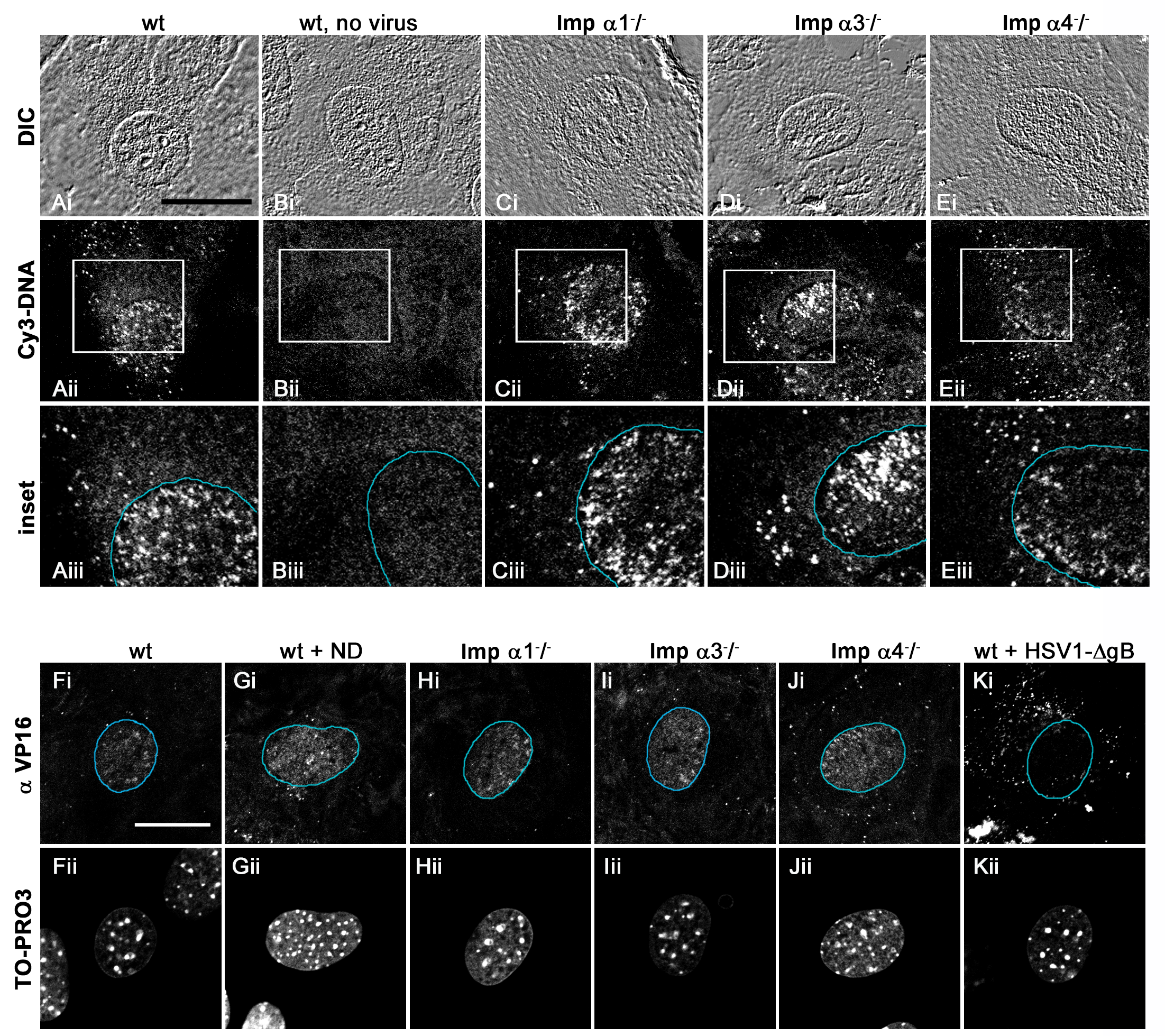

Supplement: S2 Fig — A-E: MEFwt (A, B), MEF-Impα1-/- (C), MEF-Impα3-/- (D) or MEF-Impα4-/- (E) were inoculated with HSV1(17+)Lox-CheVP26-UL37GFP (1 x 10^8^ pfu/mL, MOI of 200) or mock treated (F; MEFwt only) in the presence of cycloheximide. At 3 hpi, the cells were fixed and denatured with a mixture of 95% ethanol and 5% acetic acid, hybridized with BAC-derived HSV1(17+)Lox-Cy3-DNA (iv), and analyzed by confocal microscopy. The boxed area in ii is presented at higher magnification in iii–v. The blue lines (iv) indicate position of the nuclei as determined by DIC (i). Scale bar, 20 μm. F-K: MEFwt (F, G & K), MEF-Impα1-/- (H), MEF-Impα3-/- (I) or MEF-Impα4-/- (J) were inoculated with HSV1(17+)Lox-GFP (F-J; 1 x 10^8^ pfu/mL, MOI of 200) or with HSV1(17+)Lox-ΔgB (K) with a comparable number of viral particles in the presence of cycloheximide (F, H-K) or of cycloheximide and nocodazole (G). The cells were fixed and permeabilized with PHEMO-fix at 4 hpi, labeled with antibodies against VP16 (i), stained with TO-PRO-3 (ii; blue line in i), and analyzed by confocal microscopy. (TIF) [file ppat.1006823.s002.tif]

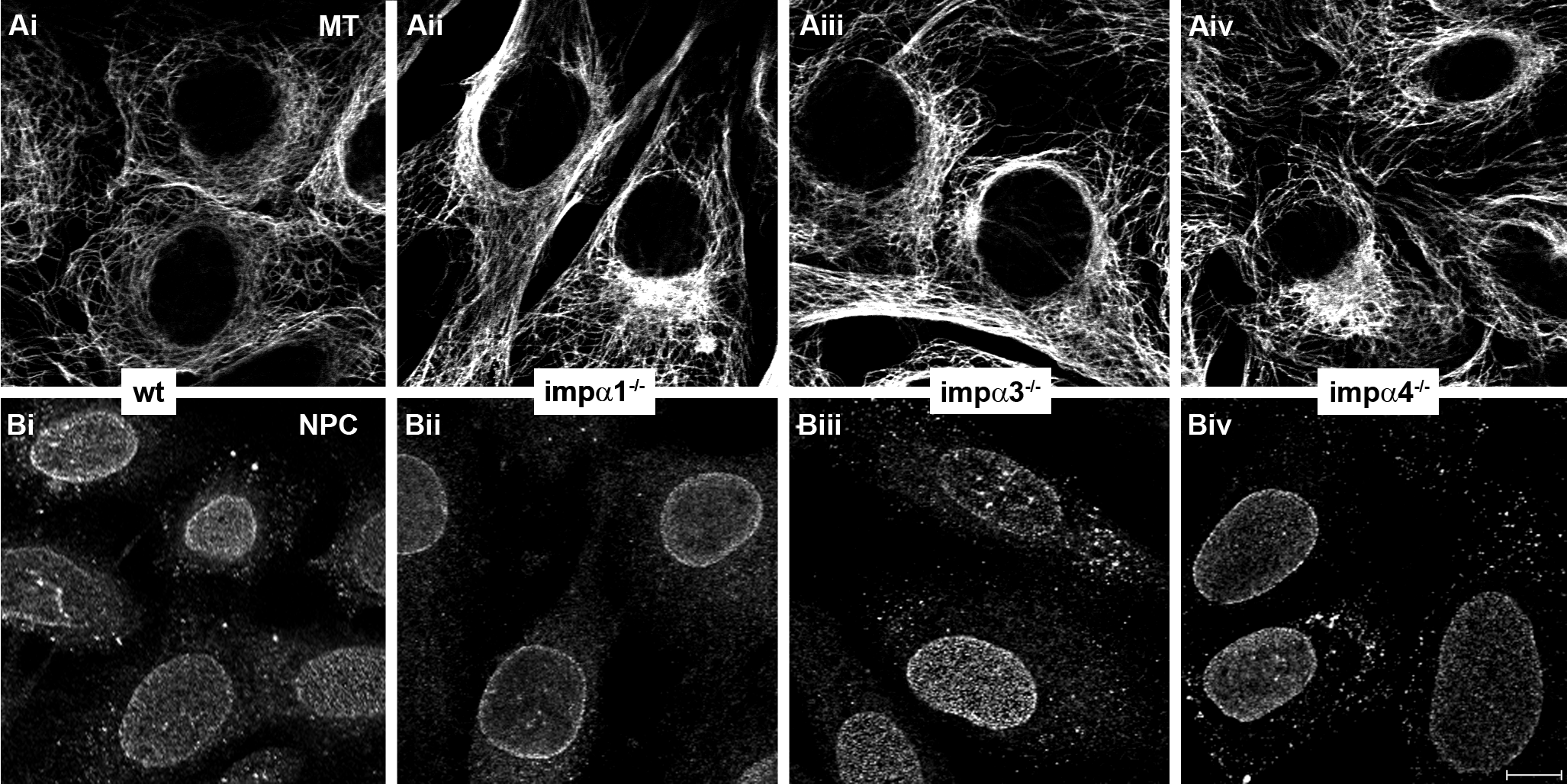

Supplement: S3 Fig — (A) Confocal microscopy of MEFwt (Ai), MEF-Impα1-/- (Aii), MEF-Impα3-/- (Aiii), and MEF-Impα4-/- (Aiv) mock treated in the presence of cycloheximide for 4 h, fixed and permeabilized with PHEMO-fix and labeled with antibodies against α tubulin. (B) Confocal microscopy of MEFwt (Bi), MEF-Impα1-/- (Bii), MEF-Impα3-/- (Biii), and MEF-Impα4-/- (Biv) inoculated with HSV1(17+)Lox-CheVP26 (5 x 10^7^ pfu/mL; MOI of 100) for 5 h in the presence of cycloheximide, fixed and permeabilized with PHEMO-fix and labeled with antibodies against NPC. Scale bar: 10 μm. (TIF) [file ppat.1006823.s003.tif]

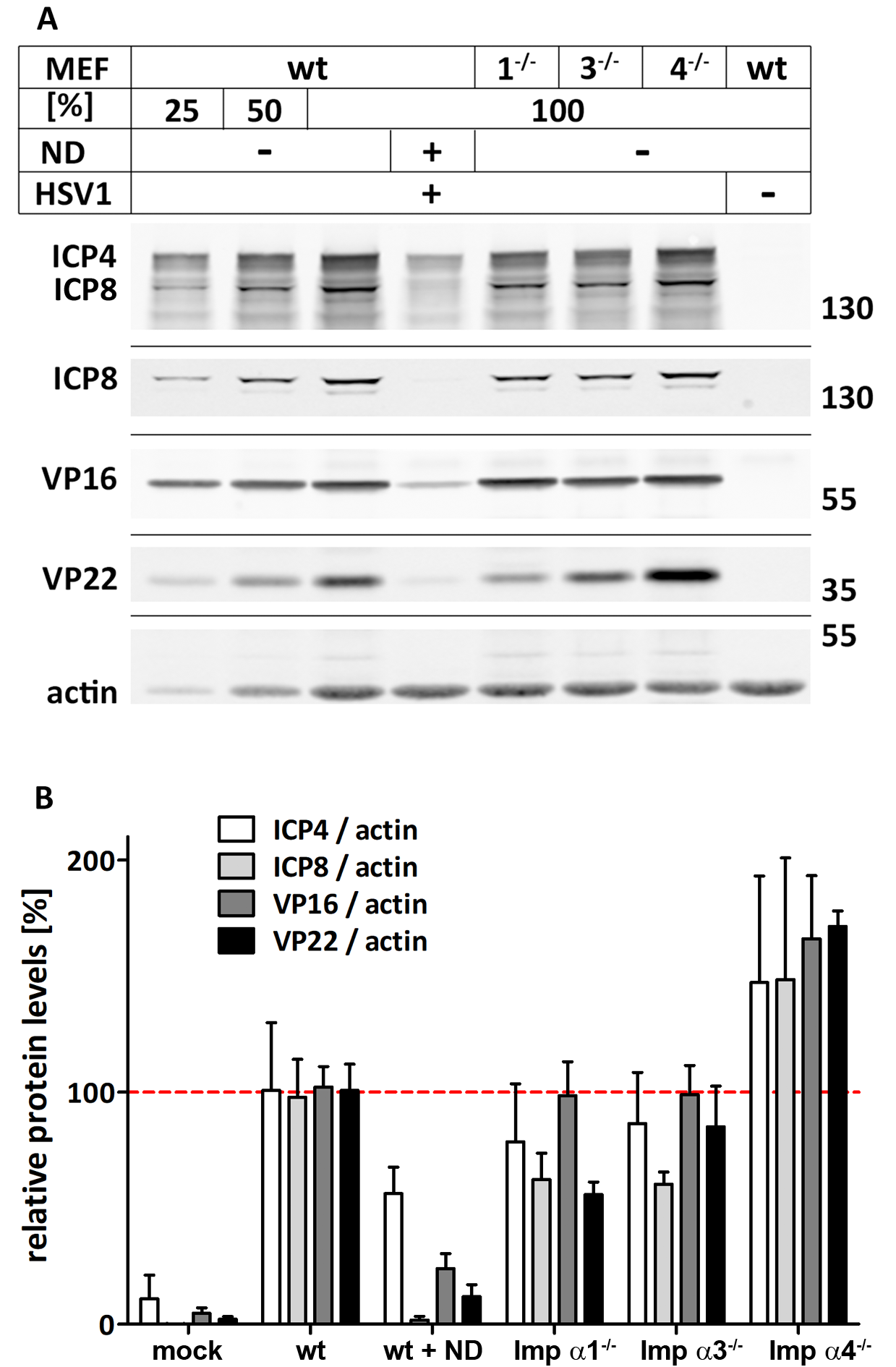

Supplement: S4 Fig — (A) MEFwt, MEF-Imp α1-/-, MEF-Imp α3-/-, or MEF-Imp α4-/- were mock infected or infected for 6 h with HSV1(17+)Lox-CheVP26 (0.5 to 1.25 x 10^6^ pfu/mL, MOI of 2 to 5 in the absence or presence of nocodazole (ND). To estimate HSV-1 expression levels upon different perturbations, 25%, 50% or 100% of a MEFwt lysates were loaded for comparison. The lysates were analyzed by immunoblot using antibodies against ICP4, ICP8, several HSV-1 structural proteins including VP16 and VP22 (pAb Remus V), or actin as a loading control. The upper part of the membrane was first incubated with anti-ICP8 (130 kDa, 2nd row) and then re-probed with anti-ICP4 (175 kDa; first row). (TIF) [file ppat.1006823.s004.tif]

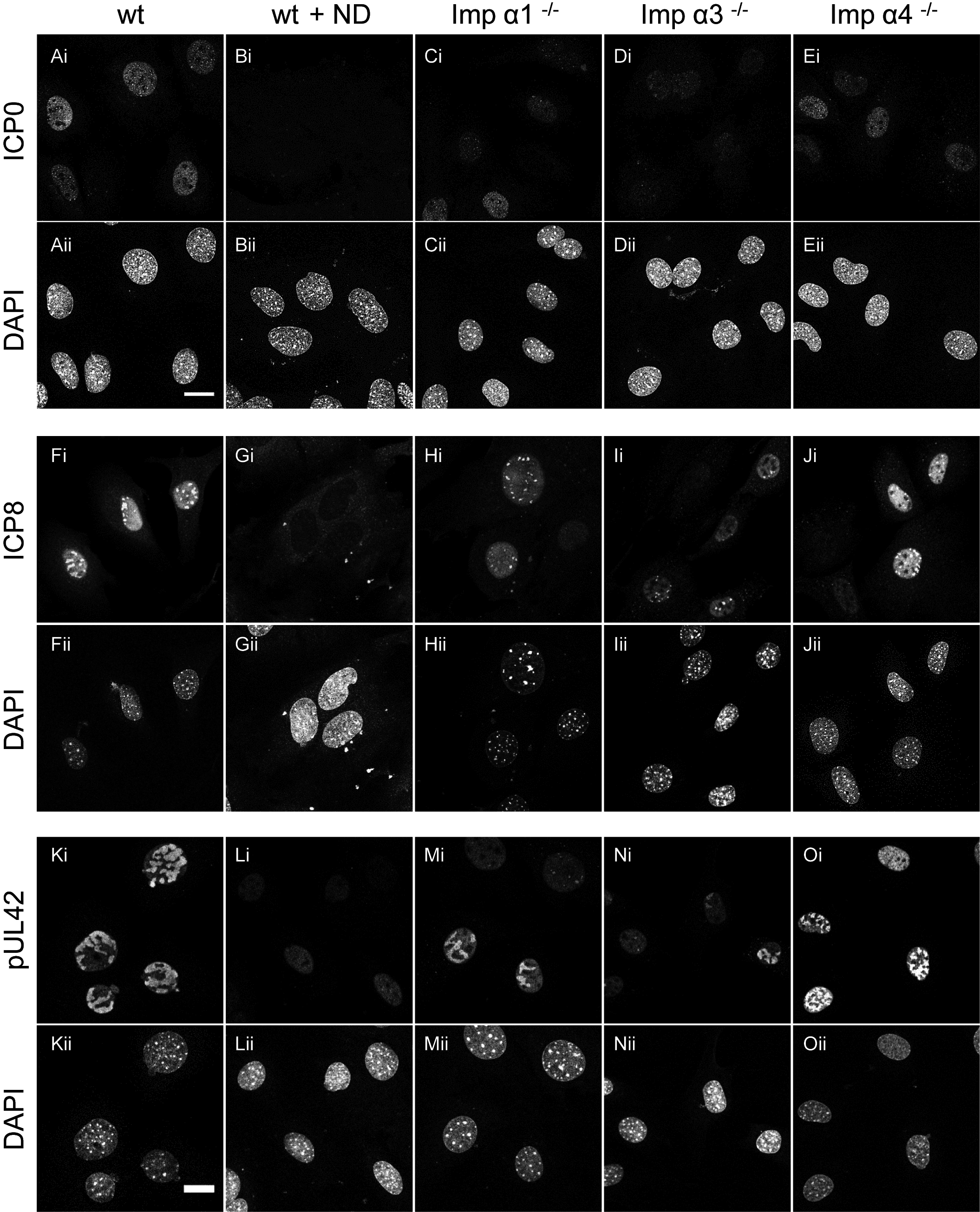

Supplement: S5 Fig — MEFwt (A, F, K), nocodazole treated MEFwt (wt + ND; B, G, L), MEF-Impα1-/- (C, H. M), MEF-Impα3-/- (D, I, N), or MEF-Impα4-/- (E, J, O) were infected with HSV1(17+)Lox-CheVP26 (0.5 to 1.25 x 10^6^ pfu/mL, MOI of 2 to 5), fixed at different times post infection with 3% PFA, permeabilized with TX-100, and labeled for ICP0 (A-E; 4 hpi), ICP8 (F-J; 6 hpi) or pUL42 (K-O; 8 hpi), and analyzed by confocal fluorescence microscopy. Scale bar 20 μm. (TIF) [file ppat.1006823.s005.tif]

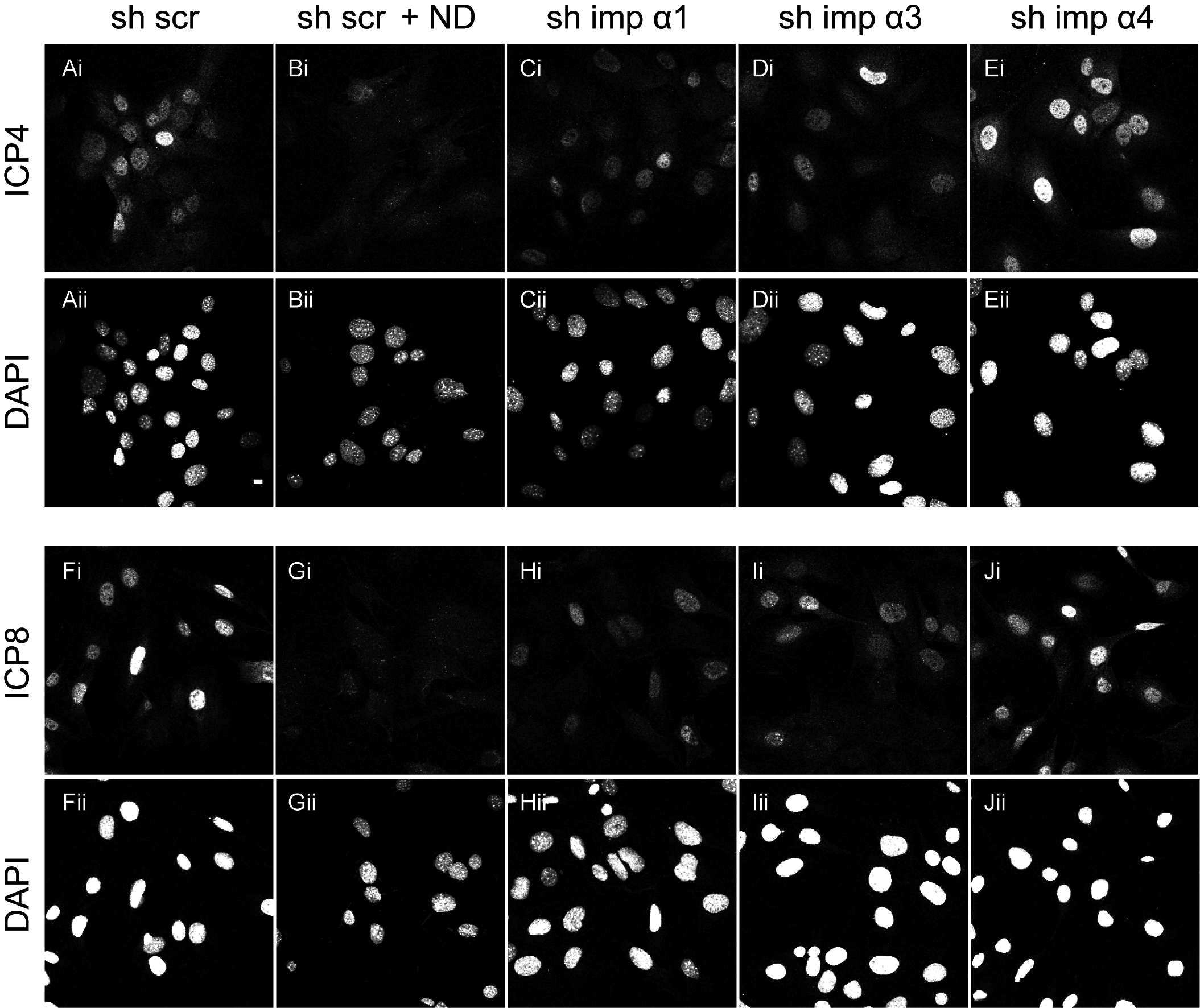

Supplement: S6 Fig — MEFwt transduced with scr shRNA (A, B, F, G) or shRNAs targeting importin α1 (C, H), α3 (D, I) or α4 (E, J) were infected with HSV1(17+)Lox-CheVP26 (0.5 to 1.25 x 10^6^ pfu/mL, MOI of 2 to 5) in the absence (A, C-E, F, H-J) or presence of nocodazole (B, G). At 4 (A-E) or 6 (F-J) hpi, cells were fixed with 3% PFA, permeabilized with TX-100, labeled with antibodies directed against ICP4 (A-E) or ICP8 (F-J), and analyzed by confocal fluorescence microscopy. (TIF) [file ppat.1006823.s006.tif]
