# Supplementary material for: Importin α1 is required for nuclear import of herpes simplex virus proteins and capsid assembly in fibroblasts and neurons
Source: PLoS Pathog. 2018 Jan 5;14(1):e1006823. doi: 10.1371/journal.ppat.1006823 (PMC5773220; doi:10.1371/journal.ppat.1006823)
Supplement: S1 Table — HeLaCNX cells were mock-treated or transfected with 50 nM of siRNA directed against different host transport factors in quadruplicate in 2 to 12 independent experiments (# of wells = 4 times # of exp.). After 3 days cells were left untreated or pre-treated with 50 μM nocodazole for 1 h and infected with 4 x 10^6^ PFU/mL of HSV1(17+)Lox-GFP for 12 h in the absence or presence of nocodazole. Cells were fixed, permeabilized, and stained with DAPI. GFP and DAPI fluorescence were measured using a fluorescence plate reader, and normalized to uninfected or DMSO treated, infected cells to express the data of different experiments as percentages (%). To reduce the impact of potential off-target effects introduced by miRNAs binding the siRNA seed region, the results were corrected using a dataset of seed region phenotypes. The seed regions of siRNAs classified by Franceschini et al. (2014) to result in off-target effects were compiled, and the mean of significantly altered seed region phenotypes were determined using a threshold of p< = 0.05 after Bonferroni correction (http://www.bioconductor.org/packages/release/bioc/manuals/scsR/man/scsR.pdf, page 26). To normalize for potential effects of RNAi on cell density, the HSV1-mediated GFP expression / cell density coefficients were calculated from the respective individual measurements: GFP—median before Bonferroni correction; GFPcorr—median after Bonferroni correction; GFPcorr/DAPI—median of individual GFPcorr/DAPI ratios. The degree of inhibition of different siRNA were then ranked; first within one transport factor and then among all transport factors investigated (av x in %), and then also expressed in absolute numbers (relative rank). (DOCX) [file ppat.1006823.s007.docx]

| **HSV1-GFP** | **siRNA #**  **(QIAGEN)** | **siRNA sequence** | **# of wells** | **# of exp.** | **GFP [%]** | **GFP^corr^ [%]** | **DAPI [%]** | **GFP^corr^/ DAPI** | **av. x**  **[%]** | **relative rank** |
| --- | --- | --- | --- | --- | --- | --- | --- | --- | --- | --- |
| **no HSV1** | N.A. | negative control for infection | 48 | 12 | 0 | 0 | 100 | 0 | 0 | **N.A.** |
| **random** | scr | positive control for infection | 48 | 12 | 100 | 100 | 100 | 1 | 100 | **N.A.** |
| **GFP** | GFP | positive control for RNAi | 48 | 12 | 19 | 19 | 96 | 0.21 | 21 | **N.A.** |
| **ND** | N.A. | without microtubules | 20 | 5 | 21 | 21 | 97 | 0.22 | 22 | **N.A.** |
| **Imp β1**  **KPNB1** | KPNB1_6 | CTGGAATCGTCCAGGGATTAA | 12 | 3 | 3 | 3 | 34 | 0.08 | 14 | **1** |
|  | KPNB1_3 | AAGGGCGGAGATCGAAGACTA | 12 | 3 | 3 | 3 | 27 | 0.11 |  |  |
|  | KPNB1_1 | TCGGTTATATTTGCCAAGATA | 12 | 3 | 5 | 5 | 42 | 0.16 |  |  |
|  | KPNB1_2 | CAAGAACTCTTTGACATCTAA | 12 | 3 | 7 | 7 | 35 | 0.20 |  |  |
| **Imp α1**  **hKPNA2** | KPNA2_5 | ACGAATTGGCATGGTGGTGAA | 16 | 4 | 4 | 4 | 24 | 0.14 | 46 | **2** |
|  | KPNA2_2 | ACCAGTGGTGGAACAGTTGAA | 16 | 4 | 37 | 37 | 78 | 0.49 |  |  |
|  | KPNA2_6 | CCGGGCTGGTTTGATTCCGAA | 8 | 2 | 65 | 65 | 90 | 0.75 |  |  |
| **Imp α6**  **hKPNA5** | KPNA5_2 | CACAATGATTATAAAGTTGTA | 16 | 4 | 12 | 12 | 65 | 0.18 | 63 | **3** |
|  | KPNA5_4 | CTCAAATTTATGTAGAGGCAA | 16 | 4 | 31 | 31 | 67 | 0.46 |  |  |
|  | KPNA5_1 | ATGGATGGATTTCAACTTTAA | 16 | 4 | 24 | 24 | 46 | 0.59 |  |  |
|  | KPNA5_3 | TAGAGTTATTAACAAATTCAA | 16 | 4 | 127 | 127 | 99 | 1.29 |  |  |
| **TNPO1**  **(KPNB2)** | TNP01_6 | CAGCATGTTAAGCCTTGTATA | 8 | 2 | 49 | 49 | 94 | 0.53 | 68 | **4** |
|  | TNP01_7 | CAGAATTGGCCTGACCTCTTA | 16 | 4 | 23 | 23 | 49 | 0.57 |  |  |
|  | TNP01_2 | CTGGAACAACTTAATCAGTAT | 8 | 2 | 53 | 53 | 80 | 0.73 |  |  |
|  | TNP01_8 | ATGCCGTTGCATCATGGATTA | 8 | 2 | 69 | 69 | 80 | 0.90 |  |  |
| **Imp α7**  **hKPNA6** | KPNA6_5 | AACCATTCTCTTACAGTTTAA | 8 | 2 | 50 | 50 | 72 | 0.65 | 86 | **5** |
|  | KPNA6_3 | CAGGTACTTATTATTGGCCAT | 8 | 2 | 58 | 58 | 69 | 0.82 |  |  |
|  | KPNA6_1 | CTGGGTGGATTACATATGATA | 8 | 2 | 77 | 77 | 89 | 0.90 |  |  |
|  | KPNA6_6 | TTGCCGAGATTACGTCTTGAA | 8 | 2 | 56 | 56 | 55 | 1.07 |  |  |
| **IPO4** | IPO4_5 | TACAGCCTATTTGCAGCCTTA | 8 | 2 | 45 | 45 | 80 | 0.57 | 90 | **6** |
|  | IPO4_1 | CTGCCTGGAGGTAGCTAGAAA | 8 | 2 | 59 | 59 | 78 | 0.76 |  |  |
|  | IPO4_6 | CCCAAGCATTTCGCTGTACAA | 8 | 2 | 80 | 80 | 63 | 1.36 |  |  |
| **Imp α3**  **hKPNA4** | KPNA4_3 | CAGCACTGAGATATATATATA | 16 | 4 | 39 | 39 | 71 | 0.60 | 90 | **7** |
|  | KPNA4_1 | CAGGTTCATCTTTGAAATCTA | 16 | 4 | 50 | 50 | 74 | 0.84 |  |  |
|  | KPNA4_4 | CACCATTAGCATCTATATCTA | 16 | 4 | 88 | 88 | 96 | 1.02 |  |  |
|  | KPNA4_2 | CTCGATGGACTAAGTAATATA | 16 | 4 | 87 | 87 | 79 | 1.15 |  |  |
| **IPO7** | IPO7_2 | CAGAAGAAGATCGCCATTGTA | 20 | 5 | 32 | 32 | 63 | 0.51 | 93 | **8** |
|  | IPO7_5 | CACCTACTACTCAATACCTTA | 20 | 5 | 119 | 119 | 90 | 1.35 |  |  |
| **Imp α4**  **hKPNA3** | KPNA3_1 | CAGCATCTTTCCCACATTCAA | 16 | 4 | 16 | 16 | 49 | 0.39 | 95 | **9** |
|  | KPNA3_2 | AAGCGTATGTTTAAACTGCAA | 12 | 3 | 93 | 93 | 92 | 0.99 |  |  |
|  | KPNA3_4 | CAGGTGTGCATTCATTACAAA | 16 | 4 | 103 | 103 | 84 | 1.16 |  |  |
|  | KPNA3_3 | CTGGATTAATTCCTATGATAA | 16 | 4 | 107 | 107 | 95 | 1.25 |  |  |
| **Imp α5**  **hKPNA1** | KPNA1_3 | CAGGTTTGTGGAGTTCCTCAA | 12 | 3 | 70 | 70 | 74 | 0.91 | 95 | **10** |
|  | KPNA1_4 | AAACCATATCCTGTAATTTAA | 12 | 3 | 77 | 77 | 101 | 0.92 |  |  |
|  | KPNA1_2 | AAAGATGAGACTAAATCTTTA | 12 | 3 | 75 | 75 | 98 | 0.93 |  |  |
|  | KPNA1_1 | ATGGGAATATACACATATTAA | 12 | 3 | 112 | 112 | 104 | 1.05 |  |  |
| **TNPO2** | TNPO2_2 | CCCGAGCATCTCTCTCTGTAA | 16 | 4 | 61 | 61 | 67 | 0.90 | 102 | **11** |
|  | TNPO2_5 | AAAGATCAGTTTCTTGTGAAA | 12 | 3 | 94 | 94 | 98 | 0.92 |  |  |
|  | TNPO2_4 | CAGGAGTGTCTCAACAACATT | 12 | 3 | 100 | 100 | 89 | 0.96 |  |  |
|  | TNPO2_3 | ATCGTGCAGGATAAACTCAAA | 12 | 3 | 110 | 110 | 79 | 1.32 |  |  |
| **RANBP5** | RANBP5_6 | CAG GTC GAA GAG TCA CTA CAA | 12 | 3 | 27 | 27 | 46 | 0.58 | 103 | **12** |
|  | RANBP5_3 | CCCGACAATGTGGTCCGGAAA | 12 | 3 | 84 | 84 | 88 | 1.02 |  |  |
|  | RANBP5_2 | CAGGATACTTGCGGCACTCAA | 12 | 3 | 71 | 71 | 79 | 1.04 |  |  |
|  | RANBP5_1 | CACGAGGCAATTAAACATGAA | 12 | 3 | 119 | 119 | 86 | 1.47 |  |  |
| **IPO13** | IPO13_1 | CAGGATGATATTCTATCCTTT | 12 | 3 | 74 | 74 | 89 | 0.83 | 128 | **13** |
|  | IPO13_5 | CTGACCAGTATGAAAGCCTAA | 12 | 3 | 68 | 42 | 43 | 0.94 |  |  |
|  | IPO13_3 | CATCATGAGGATGATCATGAA | 12 | 3 | 124 | 124 | 101 | 1.16 |  |  |
|  | IPO13_4 | CCCACGGATCAGCATCAGCAA | 12 | 3 | 106 | 106 | 58 | 2.18 |  |  |
| **IPO11** | IPO11_1 | CAGCTACAACTTTGAAGTTAA | 12 | 3 | 130 | 130 | 101 | 1.26 | 156 | **14** |
|  | IPO11_3 | CTGGAATGCAGTAGAAGTATA | 12 | 3 | 115 | 115 | 87 | 1.31 |  |  |
|  | IPO11_2 | GAGGAGATTCTTGGAAATATA | 12 | 3 | 131 | 131 | 96 | 1.44 |  |  |
|  | IPO11_4 | AACGGTTTCCATGGATCTCAA | 12 | 3 | 119 | 119 | 80 | 2.23 |  |  |
| **IPO8** | IPO8_3 | ATGCAATTAATTGATAATCAT | 16 | 4 | 71 | 71 | 94 | 0.81 | 167 | **15** |
|  | IPO8_1 | AAGAGCCTGATTGAAGATAAA | 16 | 4 | 74 | 74 | 76 | 0.92 |  |  |
|  | IPO8_2 | CCCAGTTTACTTCGGATTATA | 16 | 4 | 133 | 133 | 92 | 1.42 |  |  |
|  | IPO8_4 | CAGGTCTGTGCTACTAGACAA | 16 | 4 | 194 | 194 | 56 | 3.53 |  |  |
| **TNPO3** | TNPO3_1 | ACCGAATGTCTTAGTGAACTA | 12 | 3 | 26 | 26 | 87 | 0.30 | 179 | **16** |
|  | TNPO3_5 | CTGGGAGATCATGCAGGTTGA | 12 | 3 | 89 | 89 | 92 | 0.92 |  |  |
|  | TNPO3_4 | CAAGTGGTCATCCCTATCTTA | 12 | 3 | 99 | 99 | 93 | 1.20 |  |  |
|  | TNPO3_2 | CTGGAGATCCTTACAGTGTTA | 12 | 3 | 85 | 218 | 45 | 4.73 |  |  |
| **IPO9** | IPO9_2 | ATCAGTCATCTTGAAACAATA | 12 | 3 | 120 | 120 | 97 | 1.24 | 206 | **17** |
|  | IPO9_3 | CACAGCAAGCATGGAAAGCAA | 12 | 3 | 89 | 89 | 61 | 1.33 |  |  |
|  | IPO9_1 | CTGAATTGATTTATTATATTA | 12 | 3 | 99 | 192 | 100 | 2.28 |  |  |
|  | IPO9_4 | ATGGGTTGAGAGAATCGATAA | 12 | 3 | 89 | 235 | 82 | 3.39 |  |  |

**Supplement Table 1: Specific nuclear transport factors are required for HSV1 early gene expression.**

Targeted siRNA screen (QIAGEN) in HeLa cells for HSV1 gene expression; plate reader.

Franceschini et al. (2014) correction and Bonferroni correction; GFP/DAPI ratio

http://www.bioconductor.org/packages/release/bioc/manuals/scsR/man/scsR.pdf, page 26
