# Supplementary material for: Importin α1 is required for nuclear import of herpes simplex virus proteins and capsid assembly in fibroblasts and neurons
Source: PLoS Pathog. 2018 Jan 5;14(1):e1006823. doi: 10.1371/journal.ppat.1006823 (PMC5773220; doi:10.1371/journal.ppat.1006823)
Supplement: S2 Table — Summary of the results of the different experiments assessing different stages of the HSV-1 replication cycle in MEF-Impα1-/-, MEF-Impα3-/-, or MEF-Impα4-/- lines in comparison to the MEFwt control cell line, as well as in MEFwt or DRG neurons transduced with shRNAs targeting importin α1, α3 or α4 in comparison to scr-transduced MEFwt or neurons. (DOCX) [file ppat.1006823.s008.docx]

| **HHSV1**  **infection** | **Comparison to MEF^wt^** | | | | | | **Comparison to**  **neurons^wt^** | | |
| --- | --- | --- | --- | --- | --- | --- | --- | --- | --- |
|  | α1^-/-^ | α1^-^shRNA | α3^-/-^ | α3  shRNA | α4^-/-^ | α4  shRNA | α1^-^shRNA | α3  shRNA | α4  shRNA |
| capsids at  nuclear envelope | OK | N.D. | OK | N.D. | OK | N.D. | OK | N.D. | N.D. |
| nuclear import of VP16 | OK | N.D. | OK | N.D. | OK | N.D. | N.D. | N.D. | N.D. |
| nuclear import of  incoming genomes | OK | N.D. | OK | N.D. | OK | N.D. | N.D. | N.D. | N.D. |
| nuclear import of ICP4  immediate early gene | ↓↓ | ↓↓ | ↓↓ | ↓↓ | OK | OK | ↓↓↓ | N.D. | N.D. |
| nuclear import of ICP0  immediate early gene | ↓↓ | ↓↓ | ↓↓ | ↓↓ | OK | OK | N.D. | N.D. | N.D. |
| nuclear import of  ICP8 - early gene | ↓↓ | ↓↓ | ↓↓ | ↓↓ | ↑↑ | OK | N.D. | N.D. | N.D. |
| nuclear import of  pUL42 - early gene | ↓↓ | ↓↓ | ↓↓ | ↓↓ | OK | ↑↑ | N.D. | N.D. | N.D. |
| GFP reporter  gene expression | N.D. | N.D. | N.D. | N.D. | N.D. | N.D. | ↓↓↓ | ↓↓↓ | ↓↓ |
| nuclear capsid protein | OK | ↓↓ | OK | ↓↓ | ↑ | OK | ↓↓↓ | N.D. | N.D. |
| intracellular titer | ↓ | N.D. | delayed | N.D. | OK | N.D. | N.D. | N.D. | N.D. |
| extracellular titer | ↓ | N.D. | delayed | N.D. | OK | N.D. | N.D. | N.D. | N.D. |

**Supplementary Table S2: Summary of HSV1 replication in MEFs and neurons.**
