# Supplementary material for: Importin α1 is required for nuclear import of herpes simplex virus proteins and capsid assembly in fibroblasts and neurons
Source: PLoS Pathog. 2018 Jan 5;14(1):e1006823. doi: 10.1371/journal.ppat.1006823 (PMC5773220; doi:10.1371/journal.ppat.1006823)
Supplement: S3 Table — SIGMA TRCN numbers and sequences of the shRNAs used in this study and effect on target as assessed by immuno-blotting. Sequences used for infection assays in MEFs or DRG cells are indicated by x. (DOCX) [file ppat.1006823.s009.docx]

**Supplementary Table S3: List of tested shRNAs targeting murine importin α1, α3 or α4**

| **target** | **TRCN0000….** | **sequence**  **sense strand bold,** loop underlined | **reduction of respective target (blot)** | **used in this study for** | |
| --- | --- | --- | --- | --- | --- |
|  |  |  |  | **MEF^wt^** | **DRG** |
| **scr** | shc002 | CCGG**CAACAAGATGAAGAGCACCAA**CTCGAGTTGGTGCTCTTCATCTTGTTGTTTTT |  | x | x |
| **m Imp α1** | 093514  (#1 in S2) | CCGG**GTGTTCTCATAGATTTGTCTT**CTCGAGAAGACAAATCTATGAGAACACTTTTTG | ↓↓  (MEF, DRG, C127I) | x | x |
|  | 093515 | CCGG**CCGACTTAACAGGTTCAAGAA**CTCGAGTTCTTGAACCTGTTAAGTCGGTTTTTG | no/minor (C127I) |  |  |
|  | 093516 | CCGG**CGTGGGCTATAACCAACTATA**CTCGAGTATAGTTGGTTATAGCCCACGTTTTTG | no/minor  (C127I) |  |  |
|  | 093517 | CCGG**CCCAGCGTTTATTTCTCTCTT**CTCGAGAAGAGAGAAATAAACGCTGGGTTTTTG | no/minor  (C127I) |  |  |
|  | 093518 | CCGG**CCTGGACACTTTCAAACCTTT**CTCGAGAAAGGTTTGAAAGTGTCCAGGTTTTTG | ↓  (C127I) |  |  |
| **m Imp α3** | 093407  (#1 in S2) | CCGG**CCTGGGTTATGGTCAATTTAT**CTCGAGATAAATTGACCATAACCCAGGTTTTTG | ↓↓  (MEF, DRG) | x | x |
|  | 093405  (#2 in S2) | CCGG**GCGTGGGCTTTGACAAACATT**CTCGAGAATGTTTGTCAAAGCCCACGCTTTTTG | ↓↓  (MEF) |  |  |
| **m Imp α4** | 375209  (#1 in S2) | CCGG**GGAACGTCACATGGGTCATTG**CTCGAGCAATGACCCATGTGACGTTCCTTTTTG | ↓↓  (MEF) |  |  |
|  | 366407  (#2 in S2) | CCGG**GAGCAAATACAGATGGTTATT**CTCGAGAATAACCATCTGTATTTGCTCTTTTTG | ↓↓  (MEF) |  |  |
|  | 375274  (#3 in S2) | CCGG**CAGATGGCCACCAAGCGATAA**CTCGAGTTATCGCTTGGTGGCCATCTGTTTTTG | ↓↓  (MEF, DRG) | x | x |
